# Ferredoxin 1 is a cuproptosis-key gene responsible for tumor immunity and drug sensitivity: A pan-cancer analysis

**DOI:** 10.3389/fphar.2022.938134

**Published:** 2022-09-21

**Authors:** Longfei Yang, Yuwei Zhang, Yang Wang, Peng Jiang, Fengping Liu, Ninghan Feng

**Affiliations:** ^1^ Medical School of Nantong University, Nantong, China; ^2^ Department of Urology, Affiliated Wuxi No. 2 Hospital of Nanjing Medical University, Wuxi, China; ^3^ Wuxi School of Medicine, Jiangnan University, Wuxi, China

**Keywords:** cuproptosis, Fdx1, pan-cancer, prognosis, immune microenvironment, drug sensitivity

## Abstract

Ferredoxin 1 (FDX1) functions by transferring electrons from NADPH to mitochondrial cytochrome P450 via the ferredoxin reductase and is the key regulator in copper-dependent cell death. Although mounting evidence supports a vital role for FDX1 in tumorigenesis of some cancers, no pan-cancer analysis of FDX1 has been reported. Therefore, we aimed to explore the prognostic value of FDX1 in pan-cancer and investigate its potential immune function. Based on data from The Cancer Genome Atlas, Cancer Cell Line Encyclopedia, Genotype Tissue-Expression, Human Protein Atlas, and Gene Set Cancer Analysis, we used a range of bioinformatics approaches to explore the potential carcinogenic role of FDX1, including analyzing the relationship between FDX1 expression and prognosis, DNA methylation, RNA methylation-related genes, mismatch repair (MMR) gene, microsatellite instability (MSI), tumor mutation burden (TMB), tumor microenvironment (TME), immune-related genes, and drug sensitivity in different tumors. The results show that FDX1 was lowly expressed in most cancers but higher in glioblastoma multiforme, stomach adenocarcinoma, and uterine corpus endometrial carcinoma. Moreover, FDX1 expression was positively or negatively associated with prognosis in different cancers. FDX1 expression was significantly associated with DNA methylation in 6 cancers, while there was a correlation between FDX1 expression and RNA methylation-related genes and MMR gene in most cancers. Furthermore, FDX1 expression was significantly associated with MSI in 8 cancers and TMB in 10 cancers. In addition, FDX1 expression was also significantly correlated with immune cell infiltration, immune-related genes, TME, and drug resistance in various cancers. An experiment *in vitro* showed FDX1 is downregulated by elesclomol, resulting in inhibiting cell viability of bladder cancer, clear cell renal cell carcinoma, and prostate cancer cells. Our study reveals that FDX1 can serve as a potential therapeutic target and prognostic marker for various malignancies due to its vital role in tumorigenesis and tumor immunity.

## Introduction

The incidence and mortality of cancer have increased rapidly, seriously endangering the health and quality of human life worldwide (Sung et al., 2021). In recent years, tumor immunotherapy, such as targeting PD-1/PD-L1 or CTLA4, has emerged as a promising cancer treatment approach (Riley et al., 2019). However, cancer cells have generated complex ways to escape immune system attacks. For instance, mutation of *β2MG* can lead to *HLA* loss, resulting in lack of neoantigens present on the cell surface. Consequently, patients develop drug resistance to PD-1 due to toxic T-cell reactions (Bifulco and Urba, 2016). Moreover, the cost of cancer treatment places a huge financial burden on families and societies worldwide (Ferlay et al., 2021). Hence, there is an urgent need to identify new diagnostic biomarkers and new targets for cancer therapy.

The small iron-sulfur (Fe-S) protein encoded by ferredoxin 1 (*FDX1*) is characterized by a low redox potential, low molecular weight, and harbors at least one Fe-S cluster (Ewen et al., 2011). FDX1 can transfer electrons from NADPH to mitochondrial cytochrome P450 *via* ferredoxin reductase and is involved in the metabolism of steroids, vitamin D, and bile acid (Sheftel et al., 2010). FDX1 plays a vital role in the urogenital system; for example, *FDX1* is involved in the biosynthesis and secretion of corticosterone in mice and participates in the production of steroid hormones in human ovarian granulosa cells by regulating steroidogenic factor-1 (SF-1) (Imamichi et al., 2013; Pan et al., 2015). FDX1 is associated with the development of neonatal testis in mice and is involved in the development of polycystic ovary syndrome (PCOS) by regulating steroid metabolism and mitochondria in rat granulosa cells (Zhang et al., 2015; Wang et al., 2021). Furthermore, the deletion of *FDX1* on the long arm of chromosome 11 may be involved in the development of Lymphoproliferative disorders (LPD) (Stilgenbauer et al., 1996). Another research illustrated that polymorphisms in *FDX1* (rs10488764) were associated with the risk of IgA nephropathy (Niu et al., 2015a; Niu et al., 2015b). Growing evidence also shows that *FDX1* affects tumor development. For instance, *FDX1* is closely associated with senescence and spontaneous tumor formation in mice (Zhang et al., 2017; Zhang et al., 2020). Moreover, *FDX1* is highly expressed in human malignant melanoma cells, osteoblastic osteosarcoma tissues, and lung adenocarcinoma cells (Zhang et al., 2008; Kubista et al., 2011; Zhang et al., 2021a). Several recent articles have suggested that *FDX1* is an essential gene-regulating cuproptosis. For instance, elesclomol (ES) directly targets *FDX1* and inhibits *FDX1*-mediated Fe-S cluster biosynthesis, thereby promoting copper-dependent cell death in human breast cancer and lung adenocarcinoma cells (Tsvetkov et al., 2019). Likewise, *FDX1* is an upstream regulator of genes modified by protein lipoylation. It inhibits copper-induced cell death in human rhabdomyosarcoma and lung adenocarcinoma cells, while those effects could be reversed by elesclomol (Tsvetkov et al., 2022). However, the functional and molecular mechanisms by which *FDX1* influences tumorigenesis remain unclear.

Therefore, we used the most up-to-date data from numerous databases, including The Cancer Genome Atlas (TCGA), Cancer Cell Line Encyclopedia (CCLE), Genotype Tissue-Expression (GTEx), Human Protein Atlas (HPA), and Gene Set Cancer Analysis (GSCA), to analyze *FDX1* expression levels systematically and to evaluate their relationship with prognosis in pan-cancer. We also assessed the relationship between *FDX1* expression and DNA methylation, microsatellite instability (MSI), tumor mutation burden (TMB), and tumor microenvironment (TME) in pan-cancer. We used co-expression analysis to analyze the relationship between *FDX1* expression and RNA methylation-related genes, mismatch repair (MMR) gene, immune-related genes, and drug sensitivity in these cancers. Moreover, we investigated the biological function of *FDX1* in tumors using single-cell database and enrichment analysis and verified *in vitro* experiments with bladder cancer, clear cell renal cell carcinoma, and prostate cancer cell lines. Our results suggest that *FDX1* may serve as a therapeutic target for various cancers and plays a vital role in tumor immunity by influencing MSI, TMB, and tumor-infiltrating immune cells.

## Materials and methods

### Data collection

Gene expression, somatic mutations, and related clinical data from 33 types of cancer were downloaded from the UCSC Xena database (https://xenabrowser.net/datapages/), as well as RNA expression data from 26 types of normal tissues of GTEx. Thirty-three types of cancer cell line expression matrix of RNA expression were downloaded from the CCLE database (https://portals.broadinstitute.org/ccle/). Images of immunohistochemistry (IHC) and subcellular localization of *FDX1* were downloaded from the HPA database (https://www.proteinatlas.org/).

### FDX1 methylation profile in pan-cancer based on gene set enrichment analysis

To evaluate the *FDX1* differential degree of methylation between tumor and corresponding normal samples, the correlation between *FDX1* mRNA expression and DNA methylation in different cancer types was analyzed using the GSCA database.

### Correlation of *FDX1* expression with RNA methylation-related genes

Modification of RNA methylation, including m6A (N6-methyladenosine), m5C (methyl-5-cytosine), and m1A (N1-methyladenosine), have been shown to influence RNA function (Zhao et al., 2017). Expression profile data from TCGA was used to evaluate the correlation relationship between *FDX1* and the levels of m6A, m5C, and m1A-related genes in different cancers, including Cbl proto-oncogene like 1 (*CBLL1*), WT1 associated protein (*WTAP*), methyltransferase 3 N6-adenosine-methyltransferase complex catalytic subunit (*METTL3*), *METTL14*, RNA binding motif protein 15 (*RBM15*), *RBM15B*, zinc finger CCCH-type containing 13 (*ZC3H13*), alkB homolog 5, RNA demethylase (*ALKBH5*), FTO alpha-ketoglutarate dependent dioxygenase (*FTO*), insulin like growth factor 2 mRNA binding protein 1 (*IGF2BP1*), YTH domain containing 1 (*YTHDC1*), *YTHDC12*, YTH N6-methyladenosine RNA binding protein 1 (*YTHDF1*), *THDF2*, *YTHDF3*, NOP2/Sun RNA methyltransferase family member 7 (*NSUN7*), NOP2/Sun RNA methyltransferase 2 (*NSUN2*), *NSUN3*, *NSUN4*, *NSUN5*, *NSUN6*, tRNA aspartic acid methyltransferase 1 (*TRDMT1*), DNA methyltransferase 1 (*DNMT1*), *DNMT3*, DNA methyltransferase 3 alpha (*DNMT3A*), *DNMT3B*, NOP2 nucleolar protein (*NOP2*), tet methylcytosine dioxygenase 2 (*TET2*), tRNA methyltransferase 6 non-catalytic subunit (*TRMT6*), Aly/REF export factor (*ALYREF*), tRNA methyltransferase 61A (*TRMT61A*), *TRMT61B*, *ALKBH1* (alkB homolog 1, histone H2A dioxygenase), and *ALKBH3* (alkB homolog 3, alpha-ketoglutarate dependent dioxygenase).

### Analysis of the relationship between *FDX1* and cancer patient prognosis

Kaplan-Meier (KM) and univariate Cox regression analyses were performed to evaluate the overall survival (OS), disease-specific survival (DSS), disease-free interval (DFI), and progression-free interval (PFI) of patients from the TCGA database. Univariate Cox regression analysis was conducted using the R packages “survival” and “forestplot” to assess the relationship between *FDX1* expression and survival in pan-cancer.

### Correlation of *FDX1* expression with mismatch repair gene expression, microsatellite instability, and tumor mutation burden

TCGA expression profile data were used to assess the level of MMR gene expression, including mutL homolog 1 (*MLH1*), mutS homolog 2 (*MSH2*), *MSH3*, *MSH4*, *MSH5*, *MSH6*, PMS1 homolog 1, mismatch repair system component (*PMS1*), and *PMS2* in different cancers and to evaluate the correlation between these gene levels and *FDX1* expression. MSI scores were determined for samples in pan-cancer based on somatic mutation data downloaded from the TCGA database. Data of varscan2 variant aggregation and masking was downloaded from the UCSC Xena database and used a Perl script to calculate TMB scores. Spearman’s rank correlation coefficient was used to analyze the relationship of *FDX1* expression with that of MMR gene, MSI, and TMB.

### Relationship between *FDX1* expression and immunity

The relationship between *FDX1* expression and the TME was studied using the ESTIMATE algorithm. This algorithm was used to calculate the immune and stromal scores for each pan-cancer tumor sample. The relationship between *FDX1* expression and these scores was evaluated according to the degree of immune infiltration using the R packages “estimate” and “limma.” We downloaded the immune cell infiltration score of TCGA from the CIBERSORT database (https://cibersortx.stanford.edu/) and used CIBERSORT to calculate the relative scores for 22 immune cells in pan-cancer. Spearman’s rank correlation coefficient was used to analyze the correlation between *FDX1* levels and the infiltration level of each immune cell in cancer. In addition, we explored the association between *FDX1* expression and immune-related genes, which are chemokine, chemokine receptor, major histocompatibility complex (MHC), immunosuppressive, immunostimulatory, and immune checkpoint-related genes, in pan-cancer, using the the R package “limma.” The “reshape2” and “RColorBrewer” packages were used to visualize the results.

### Drug sensitivity analysis

The relationship between drug sensitivity and *FDX1* mRNA expression was evaluated. We performed drug screening data using the GSCA database, which integrated over 10,000 genomic data in pan-cancer from TCGA and over 750 small-molecule drugs from the Genomics of Drug Sensitivity in Cancer (GDSC) database.

### Single-cell sequencing data analysis

To validate the different functions of *FDX1* in cancer cells at the single-cell level, we downloaded the relevant data from the CancerSEA database and created a heatmap. Furthermore, the t-SNE diagrams of all individual cells were also obtained from CancerSEA.

### Gene set enrichment analysis and protein-protein interaction analysis

Gene Set Enrichment Analysis (GSEA) was conducted to explore the biological functions of *FDX1* across the pan-cancer. The Kyoto Encyclopedia of Genes and Genomes (KEGG) gene sets were downloaded from the GSEA website (https://www.gsea-msigdb.org/gsea/downloads.jsp) and used the R packages “limma,” “org.Hs.eg.db,” “clusterProfiler,” and “enrichplot” to investigate the functions of *FDX1*. We used the String database to create an interaction network for *FDX1*. A protein was considered to interact with *FDX1* in the network if its interaction score exceeded 0.4. Active interaction sources included text mining, experiments, databases, co-expression, neighborhood, gene fusion, and co-occurrence.

### Cell culture and chemicals

The immortalized normal human urothelial cell line SV-HUC-1 and human bladder urothelial carcinoma (BLCA) cell lines T24 and 5637, and human proximal tubular epithelial cell line HK-2 and human clear cell renal cell carcinoma (ccRCC) cell lines 786-O and caki-1, and human normal prostatic epithelial cells RWPE-1 and human prostate cancer (PCa) cell lines PC-3 and DU145 were all purchased from Procell Life Science and Technology Co., Ltd. (Wuhan, China). SV-HUC-1 and PC-3 cells were cultured in Ham’s F-12K medium (Procell) supplemented with 10% fetal bovine serum (FBS) and 1% penicillin/streptomycin. T24, HK-2, caki-1, and DU145 cells were cultured in DMEM medium (Procell) supplemented with 10% FBS and 1% penicillin/streptomycin. RWPE-1 cells were cultured in complete medium of human prostatic epithelial cells (Procell). Furthermore, 5637 and 786-O cells were cultured in RPMI 1640 medium (Procell) supplemented with 10% FBS and 1% penicillin/streptomycin. All the cell lines were cultured in an incubator with 5% CO_2_ at 37 °C. Elesclomol (Synta Pharmaceuticals, Lexington, MA, United States) was diluted in a cell culture medium at 100 nmol/L concentration.

### Reverse transcription-quantitative polymerase chain reaction

Total RNA from mentioned above cell lines was isolated using the TransZol Up Plus RNA kit (TransGen Biotechnology, Beijing, China). RNA concentration was measured using NanoDrop 2000 (Thermo Fisher Scientific, Waltham, MA, United States). Next, cDNA was obtained using reverse transcription (RT). Finally, RT-quantitative polymerase chain reaction (qPCR) was conducted to investigate the expression of *FDX1* using Green qPCR SuperMix (TransGen Biotechnology). The specific primers in our study were as follows: *FDX1*, F-5′-CGATGGCATCAAGGTCTTTCCC-3′, and R-5′-CAGCAGGAGTTTCATGCGGAAC-3′; *β-Actin*, F-5′-GACGTGGACATCCGCAAAG-3′, and R-5′-CTGGAAGGTGGACAGCGAGG-3′. Finally, the relative expression of *FDX1* was calculated using the 2−^ΔΔ^CT method.

### Western blotting

Whole-cell lysates were extracted using RIPA buffer (Applygen Technologies Inc., Beijing, China) supplemented with a protease inhibitor cocktail (GlpBio Technology, Shanghai, China). The protein concentration was measured using a BCA protein assay kit (Elabscience Biotechnology Co., Ltd., Wuhan, China). Cell lysates were performed by sodium dodecyl sulfate-polyacrylamide gel electrophoresis (SDS-PAGE), and the protein bands were electrophoretically transferred onto PVDF membranes. Subsequently, non-specific antigen binding was blocked using 5% skim milk. The membranes were incubated at 4°C overnight with primary antibodies (1:1,000-diluted FDX1; Proteintech Group, Inc., Wuhan, China). Membranes were incubated the next day with 1:5,000-diluted secondary antibodies at room temperature for 1 h (Proteintech Group). Enhanced chemiluminescence was used to visualize the image (Proteintech Group). Antibody against β-actin was used as an internal reference (Proteintech Group).

### CCK-8 assay

After treatment with elesclomol for 16 h, the cells were transferred to 96-well plates (100 μL cell suspension per well) at a density of 3,000 cells/well in triplicate for each group and were incubated in a humidified incubator. CCK-8 reagent (Transgene Biotech) was added to each well, and the cells were incubated for an additional hour. A microplate reader (Biotek Gen5, Santa Clara, CA, United States) was used to measure the absorbance (optical density [OD] value) at 450 nm. OD values were measured at 0, 24, 48, and 72 h.

### Caspase3/7 activity assay

Caspase3/7 activity of the cells was measured using a caspase3/7 live-cell fluorescence real-time detection kit (Beijing BioRab Technology Co. Ltd., China) according to the manufacturer’s protocol. Cells were treated with elesclomol and incubated at 37°C and 5% CO_2_ for 16 h. Next, the cells were washed twice with fresh medium and caspase3/7 activity detection reagent working solution added to each well at a concentration of 1 μl/ml. After incubating at 37°C and 5% CO_2_ for 30 min, fluorescence was measured using a microplate reader (Biotek Gen5).

### Statistical analysis

All gene expression data were normalized using a log2 transformation. The correlation analysis between the two variables used Spearman’s or Pearson’s test; *p* < 0.05 was considered significant. Comparison of difference between adjacent normal and tumor tissues used Wilcoxon test; *p* < 0.05 indicated the statistical significance. The Kaplan-Meier curves and univariate Cox proportional hazard regression models were used for all survival analyses. Statistical analyses of the bioinformatics results were performed using R software (Version 4.1.2). The statistical significance of the *in vitro* data was determined using GraphPad Prism (version 9; GraphPad Inc., La Jolla, CA, United States).

## Results

### Differential expression of *FDX1* between tumor and normal tissue samples

**FIGURE 1:**
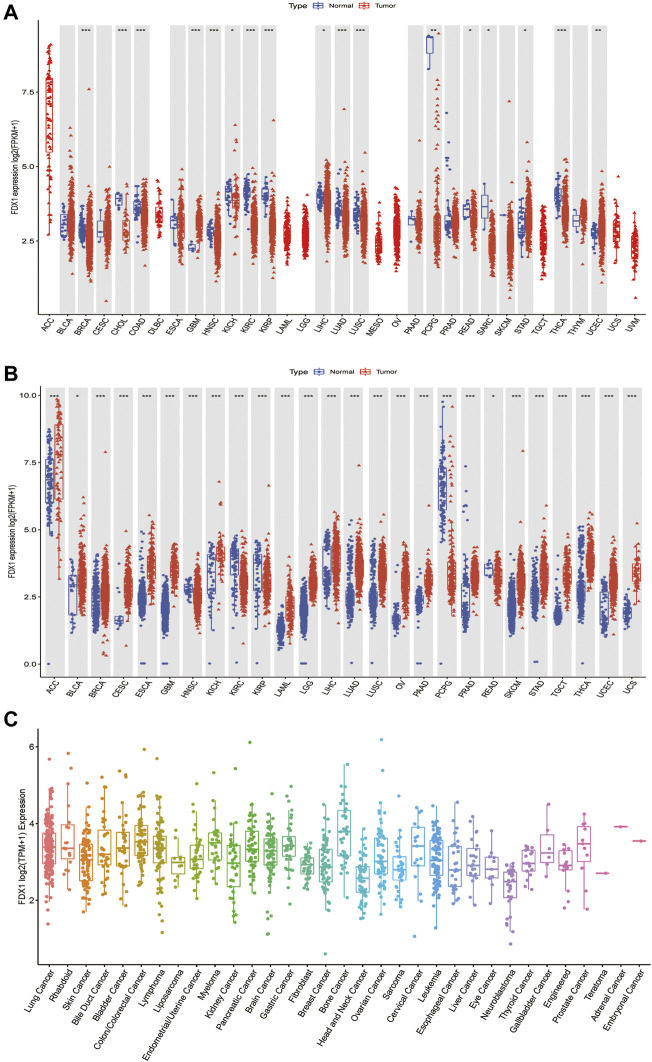
Differential expression of *FDX1*. **(A)** Comparison of *FDX1* expressions between tumors and normal samples in the TCGA database. **(B)** Comparison of *FDX1* expressions between tumors and normal samples in the TCGA and the GTEx databases. **(C)** *FDX1* expression in tumor cell lines. Log2 (FPKM+1) and log2 (TPM+1) are used for the logarithmic scale. *p < 0.05, **p < 0.01, ***p < 0.001.

To better understand *FDX1* expression in various cancer types, we first analyzed its expression in 33 cancers using TCGA data. Excluding those cancers without corresponding normal samples, significant differences in *FDX1* expression were found between tumor and normal tissues in 17 types of cancer. *FDX1* was lowly expressed in breast invasive carcinoma (BRCA), cholangiocarcinoma (CHOL), colon adenocarcinoma (COAD), head and neck squamous cell carcinoma (HNSC), kidney chromophobe (KICH), kidney renal clear cell carcinoma (KIRC), kidney renal papillary cell carcinoma (KIRP), liver hepatocellular carcinoma (LIHC), lung adenocarcinoma (LUAD), lung squamous cell carcinoma (LUSC), pheochromocytoma, and paraganglioma (PCPG), rectum adenocarcinoma (READ), sarcoma (SARC), and thyroid carcinoma (THCA), while highly expressed in glioblastoma multiforme (GBM), stomach adenocarcinoma (STAD), and uterine corpus endometrial carcinoma (UCEC) (Figure 1A, Supplementary Table S1). Due to some cancers having very few normal samples in the TCGA database, we used data from the GTEx database, which included diverse tissues from healthy persons. When we combined data from TCGA and GTEx databases, we found that the expression of *FDX1* was highly different in 26 cancers. FDX1 was lower in HNSC, KIRC, KIRP, PCPG, and READ but was higher expressed in other 21 tumor types (Figure 1B, Supplementary Table S1). Moreover, we also investigated the expression of *FDX1* in different cancer cell lines using the CCLE database (Figure 1C).

Next, to evaluate *FDX1* expression at the protein level, we analyzed the results provided in the HPA database. As shown in Supplementary Figures S1A–F, FDX1 displayed medium-to-high expression positivity in the normal adrenal gland, testis, placenta, and kidney tissues. On the contrary, *FDX1* displayed weak-to-moderate cytoplasmic positivity in thyroid cancer, colorectal cancer, liver cancer, and melanoma. Furthermore, normal liver tissues did not reveal *FDX1* staining, while tumor liver tissues showed moderate staining. Normal breast and lung tissues, and lung and breast tumor tissues did not detect with *FDX1* staining. We also analyzed the subcellular location of *FDX1* in the HPA database. The protein of *FDX1* was mainly localized to the mitochondria and was enhanced in cell lines of A-431 (human epidermoid carcinoma cells), U-2 OS (human osteosarcoma cells), U-251 MG (human glioma cells), and particularly in CACO-2 (human colorectal adenocarcinoma cells) (Supplementary Figures S2A–D). Taken together, compared to adjacent normal tissues, *FDX1* expression is dysregulated in 29 cancers, indicating that it may serve as an oncogene or suppressor in these cancers. In subsequent analysis, we only focus on those cancer types sensitive to the *FDX1* gene expression.

### Ferredoxin 1 is associated with expression levels of DNA methylation and RNA methylation-related genes across cancers

**FIGURE 2:**
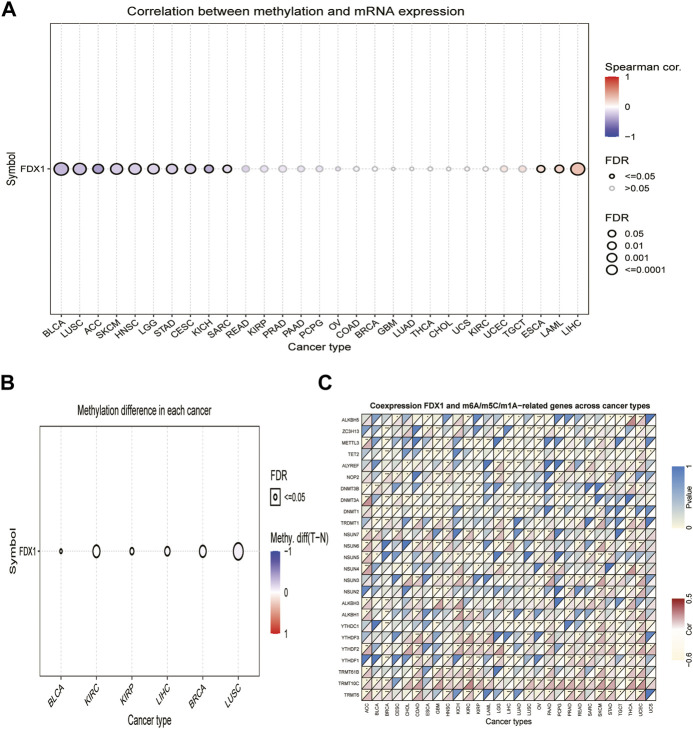
Association between *FDX1* expression and DNA methylation, and m6A, m5C, m1A-related genes in pan-cancer. **(A)** Correlation between methylation and mRNA expression of *FDX1*. **(B)** Methylation difference of *FDX1* mRNA in different cancers. **(C)** Heatmap illustrating the relationship between *FDX1* and m6A, m5C, and m1A-related genes. The top left triangle represents the *p*-value, and the bottom right triangle represents the correlation coefficient. *p < 0.05, **p < 0.01, ***p < 0.001.

DNA methylation alterations have been observed in various cancers and are considered a cause of carcinogenesis (Klutstein et al., 2016). We calculated the correlation between promoter methylation and mRNA expression of *FDX1* using the GSCA database. Our data revealed a correlation between *FDX1* expression and DNA methylation in 13 tumors (Figure 2A, Supplementary Table S1). The relationship between *FDX1* expression and promoter methylation levels was significantly positive in BRCA, KIRP, LIHC, and LUSC, while negative in BLCA and KIRC (Figure 2B).

Modification of RNA methylation, including m6A, m5C, and m1A, results in different outcomes that influence RNA functions and may contribute to tumorigenesis (Zhao et al., 2017). We, therefore, evaluated the relationship between *FDX1* and levels of RNA methylation-related genes. Our results illustrated that *FDX1* expression was positively correlated with *YTHDF2* and *TRMT10C* expression in most cancers, except LIHC and testicular germ cell tumors (TGCT). Likewise, *FDX1* expression was positively correlated with *NSUN6* levels in 10 tumors, except for GBM, KIRC, KIRP, brain lower grade glioma (LGG), and ovarian serous cystadenocarcinoma (OV). In addition, *FDX1* expression was positively correlated with *YTHDF1* in 11 tumors but not in BRCA, KIRP, LIHC, and LUAD (Supplementary Table S1). As shown in Figure 2C, *FDX1* expression correlated negatively with that of *TET2* and *DNMT1* in BRCA, esophageal carcinoma (ESCA), GBM, HNSC, LIHC, LUAD, LUSC, and PCPG and correlated positively in skin cutaneous melanoma (SKCM). Contrasting results were found for the expression of *TRMT6*, which was positively correlated with *FDX1* expression in 14 tumors, but not in KIRP, LIHC, and TGCT. These results show that the altered epigenetic status of *FDX1* may contribute to tumorigenesis.

### Prognostic significance of Ferredoxin 1

**FIGURE 3:**
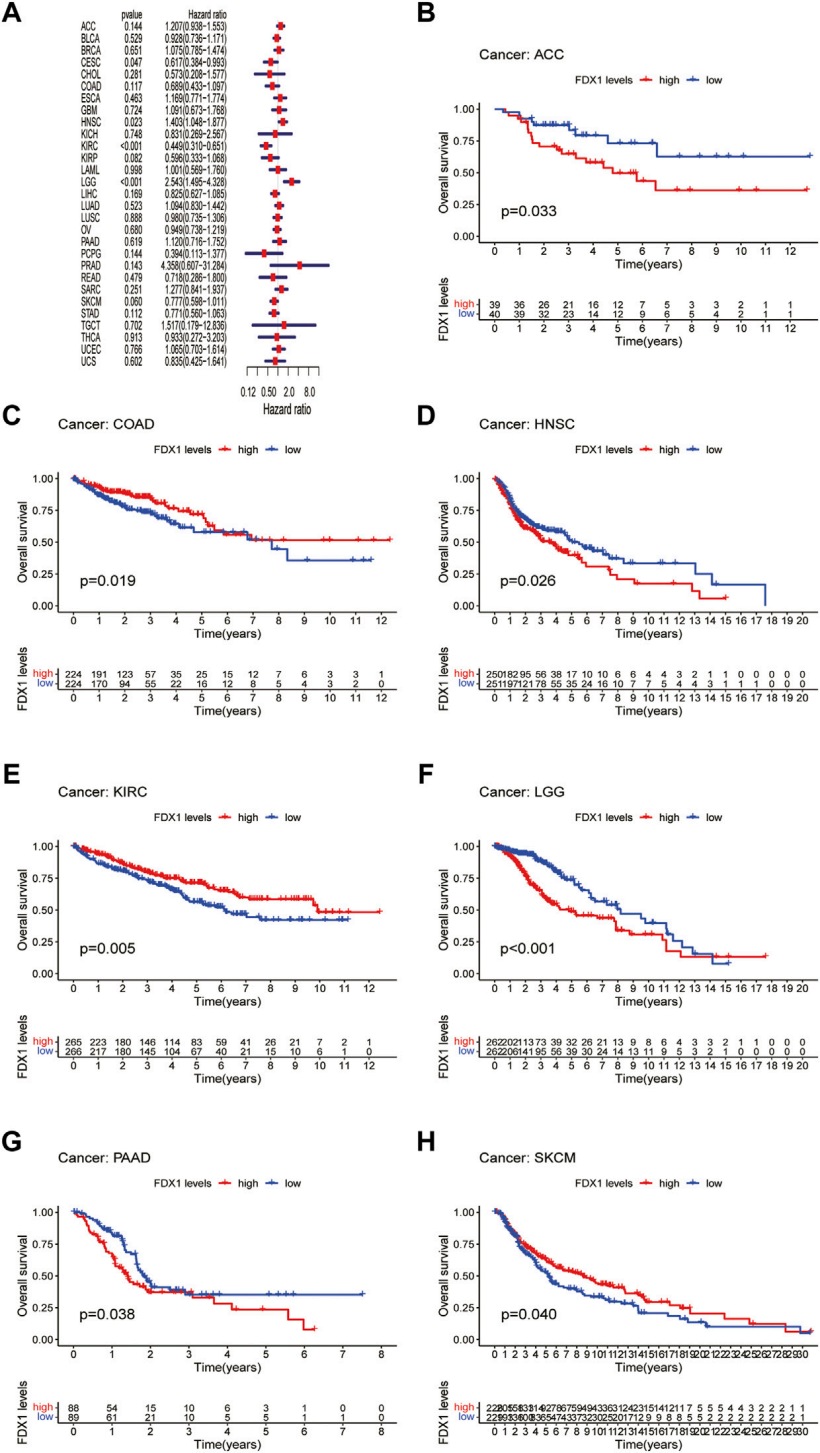
Association between *FDX1* expression and overall survival (OS). **(A)** Forest plot shows the univariate cox regression results for the association between *FDX1* expression and OS in pan-cancer. **(B–H)** Kaplan-Meier analysis of the association between *FDX1* expression and OS.

**FIGURE 4:**
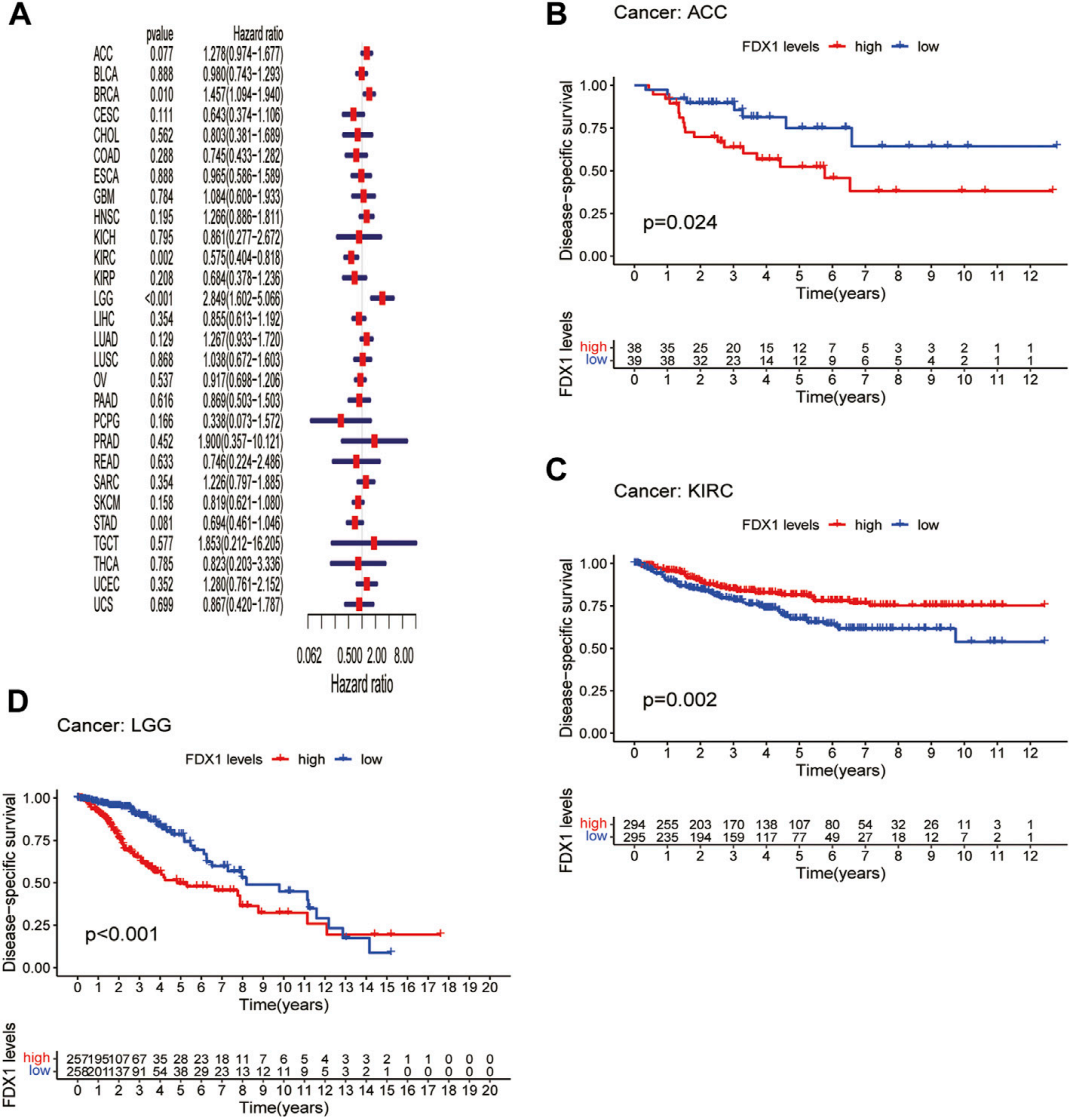
Association between *FDX1* expression and disease-specific survival (DSS). **(A)** Forest plot shows the univariate cox regression results for the association between *FDX1* expression and DSS in pan-cancer. **(B–D)** Kaplan-Meier analysis of the association between *FDX1* expression and DSS.

Next, we evaluated the prognostic value of *FDX1* expression in pan-cancer, including OS, DSS, DFI, and PFI analyses based on the TCGA database. Analysis of OS by Cox regression indicated that high expression of *FDX1* was associated with longer survival times in cervical squamous cell carcinoma and endocervical adenocarcinoma (CESC) and KIRC, but not in HNSC and LGG (Figure 3A). Furthermore, OS by KM analysis revealed that *FDX1* was a risk factor for patients with adrenocortical carcinoma (ACC), HNSC, LGG, and pancreatic adenocarcinoma (PAAD) and was a protective factor for patients with COAD, KIRC, and SKCM (Figures 3B–H). Since non-tumor-related factors may lead to death during follow-up, we analyzed the relationship between *FDX1* expression and DSS in pan-cancer. As shown in Figure 4A, DSS by Cox regression analysis indicated that *FDX1* was a risk factor for patients with BRCA and LGG, and a protective factor for patients with KIRC. KM analysis of DSS illustrated that high *FDX1* expression corresponded with longer DSS in KIRC and with poor DSS in ACC and LGG (Figures 4B–D).

**FIGURE 5:**
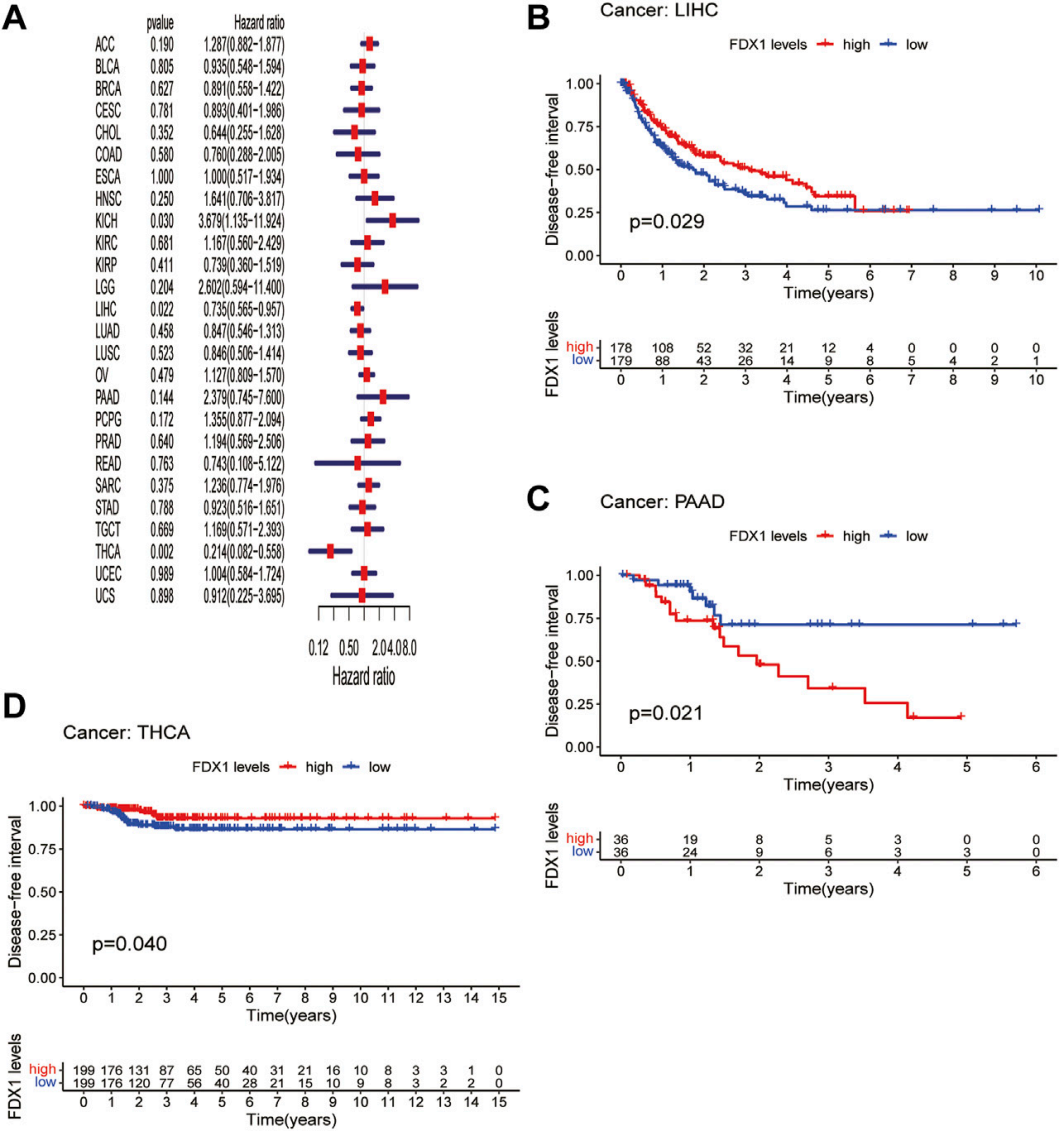
Association between *FDX1* expression and disease-free interval (DFI). **(A)** Forest plot shows the univariate cox regression results for the association between *FDX1* expression and DFI in pan-cancer. **(B–D)** Kaplan-Meier analysis of the association between *FDX1* expression and DFI.

**FIGURE 6:**
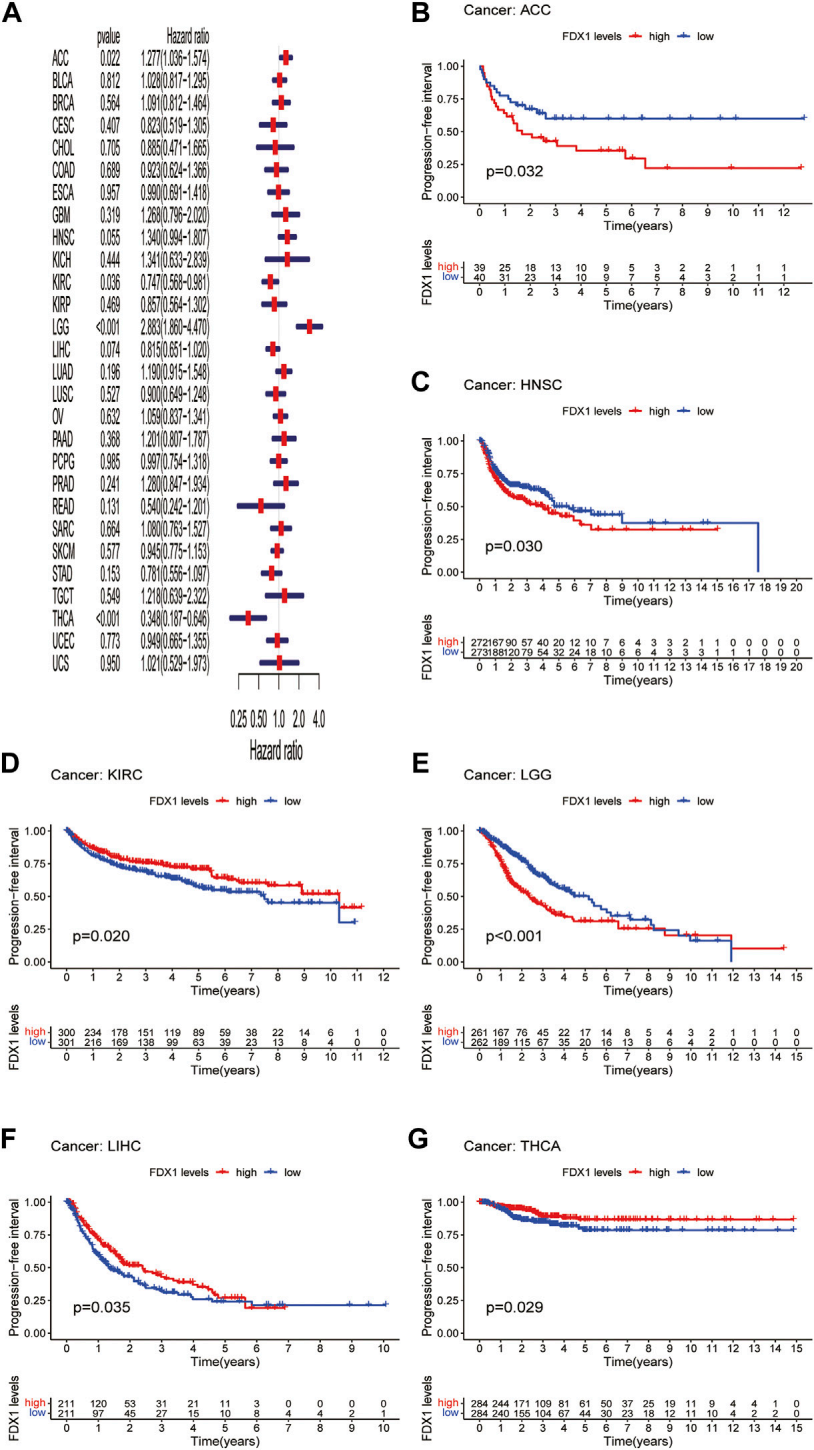
Association between *FDX1* expression and progression-free interval (PFI). **(A)** Forest plot shows the univariate cox regression results for the association between *FDX1* expression and PFI in pan-cancer. **(B–G)** Kaplan-Meier analysis of the association between *FDX1* expression and PFI.

DFI is used to evaluate radical surgery outcomes and generally represents the time from treatment to recurrence. We performed DFI analysis using Cox regression and found that *FDX1* was a protective factor for patients with LIHC and THCA but not for those with KICH (Figure 5A). Similarly, analysis of DFI by KM indicated that high *FDX1* expression was associated with a poor prognosis in PAAD patients, while it indicated a better prognosis in LIHC and THCA patients (Figures 5B–D). In addition, we assessed the relationship between *FDX1* expression and PFI, a measure of how well cancer responds to palliative care. PFI analyzed by Cox regression revealed that *FDX1* also acted as a risk factor in patients with ACC and LGG but as a protective factor in patients with KIRC and THCA (Figure 6A). Likewise, KM analysis of PFI showed that *FDX1* was a protective factor in patients with KIRC, LIHC, and THCA but was a risk factor in patients with ACC, HNSC, and LGG (Figures 6B–G). These data suggest that *FDX1* expression is significantly associated with patient prognosis in multiple cancer types, particularly in ACC, LGG, KIRC, and THCA.

### Correlation of *FDX1* expression levels with mismatch repair gene expression, microsatellite instability, and tumor mutation burden

**FIGURE 7:**
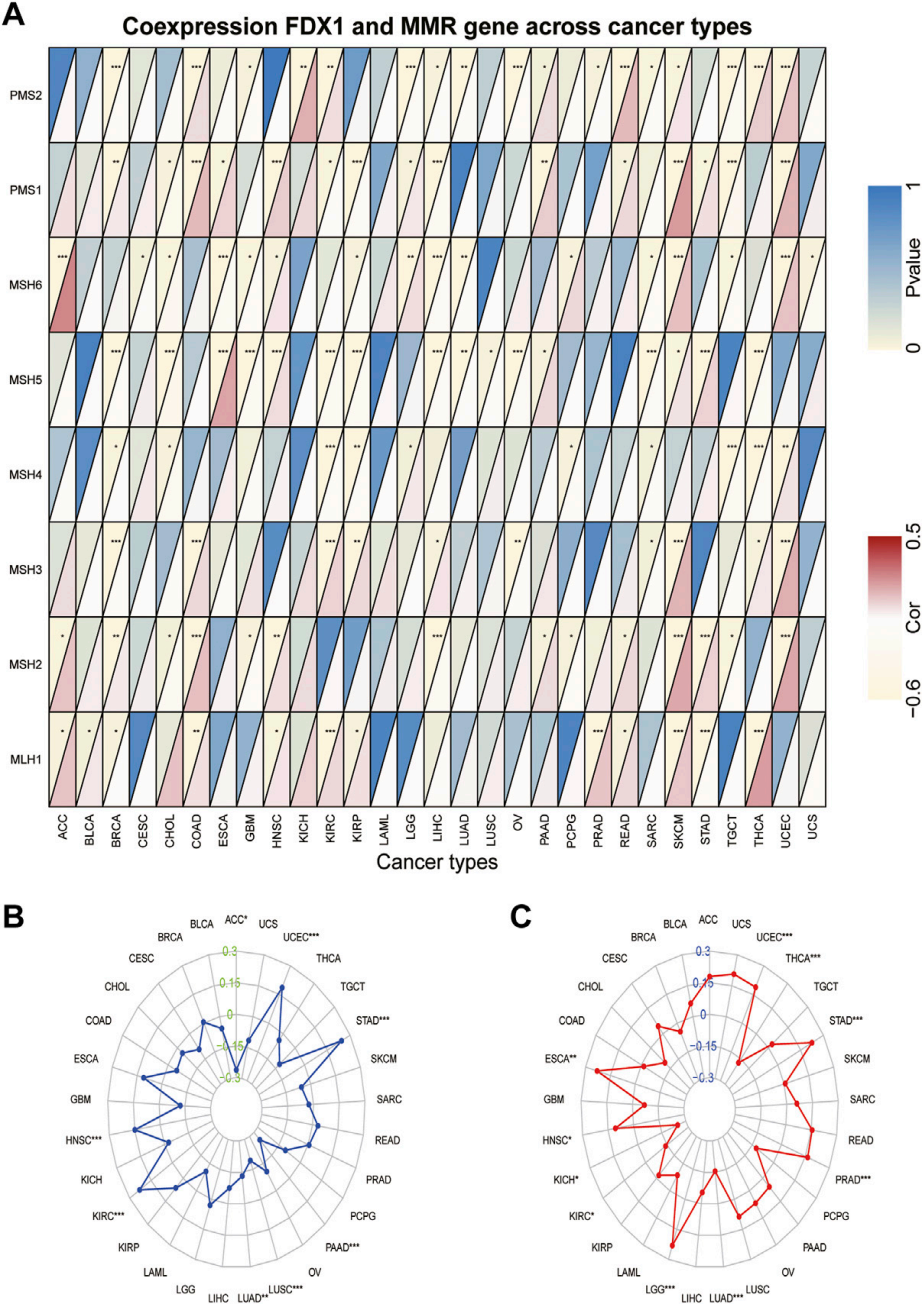
Association between *FDX1* expression and mismatch repair (MMR) gene, microsatellite instability (MSI), and tumor mutation burden (TMB) in pan-cancer. **(A)** Heatmap illustrating the relationship between *FDX1* and MMR gene. The top left triangle represents the *p*-value, and the bottom right triangle represents the correlation coefficient. *p < 0.05, **p < 0.01, ***p < 0.001. **(B)** Correlation between *FDX1* expression and MSI across cancers. **(C)** Correlation between *FDX1* expression and TMB across cancers. The value of black represents the range, and the curves of blue and red represent the correlation coefficients.

Tumors with defects in the MMR system are vulnerable to mutations in microsatellites (Jiang et al., 2021), causing high levels of MSI, leading to the accumulated mutation loads in cancer-related genes and the aggravation of the TMB (Picard et al., 2020). Hence, we analyzed the relationship between *FDX1* expression and the expression levels of the MMR gene, including *MLH1*, *MSH2*, *MSH3*, *MSH4*, *MSH5*, *MSH6*, *PMS1*, and *PMS2*. We found that *FDX1* expression was significantly correlated with that of *PMS2* in 17 types of cancer (Supplementary Table S1). Specifically, it was negatively correlated in BRCA, GBM, LGG, LIHC, LUAD, OV, prostate adenocarcinoma (PRAD), SARC, and TGCT but was positively correlated in the other eight cancers (Figure 7A). Likewise, *FDX1* expression was significantly negatively correlated with *MSH5* in 11 tumors but not in ESCA, HNSC, PAAD, SKCM, and STAD. In contrast, *MLH1* was significantly positively correlated with *FDX1* expression in 10 tumors but not in BRCA and STAD. We also analyzed the relationship between the expression of *FDX1* and MSI and TMB. *FDX1* expression was significantly correlated with MSI in 8 tumors: it was positively correlated in HNSC, KIRC, STAD, and UCEC, and negatively in the other four cancers (Figure 7B). As shown in Figure 7C, *FDX1* expression was highly correlated with the TMB in 10 types of tumors, particularly in LGG, LUAD, PRAD, STAD, THCA, and UCEC. In summary, these results indicate that *FDX1* may mediate tumorigenesis by regulating DNA damage.

### Relationship between *FDX1* expression and tumor microenvironment

**FIGURE 8:**
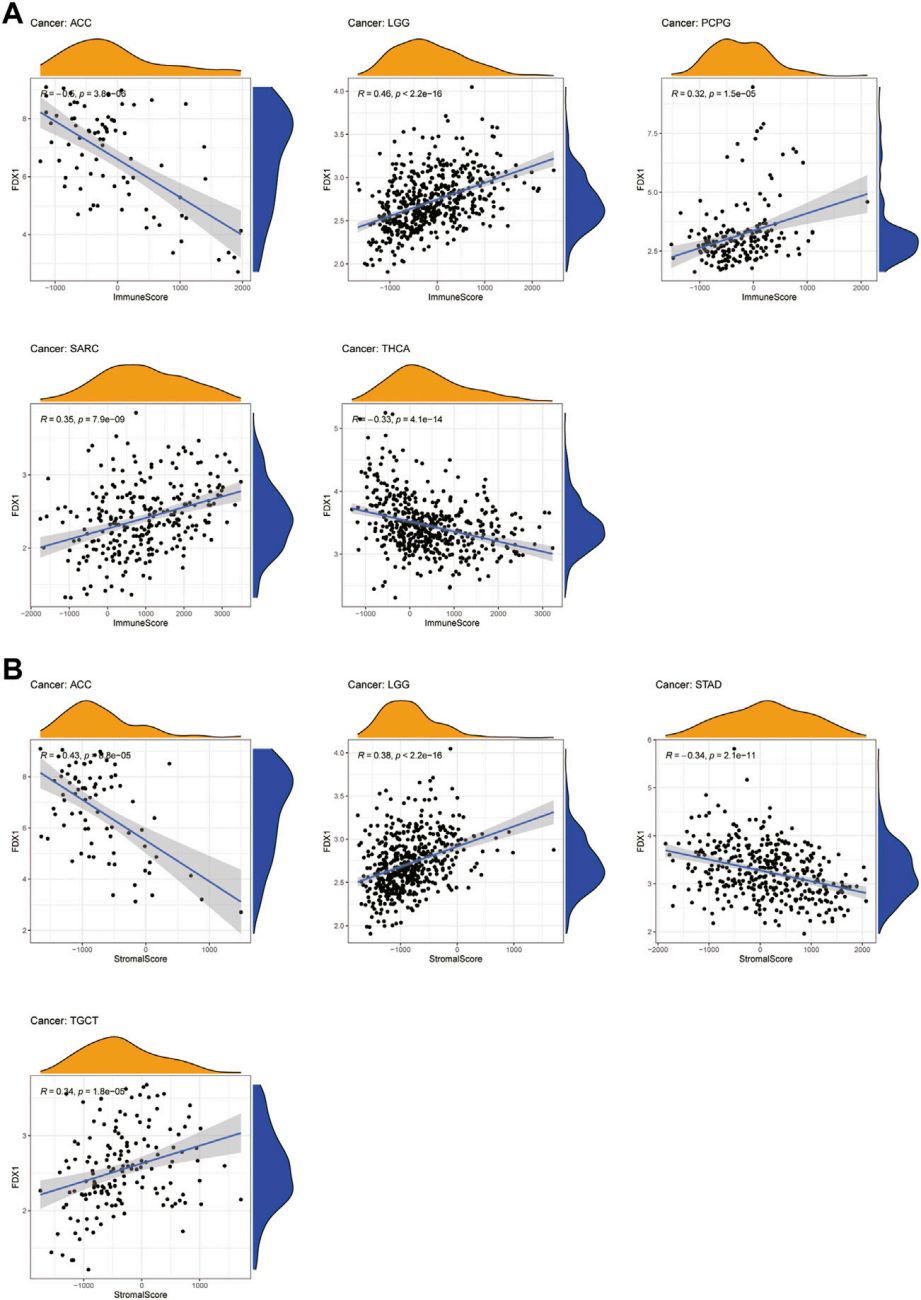
Seven tumors with the highest correlation coefficients between *FDX1* expression and the tumor microenvironment. **(A)** Correlation between *FDX1* and immune scores in ACC, LGG, PCPG, SARC, and THCA. **(B)** Correlation between *FDX1* and stromal scores in ACC, LGG, STAD, and TGCT.

An increasing number of reports have indicated that the TME plays a vital role in tumor occurrence and development (Xiao and Yu, 2021). Therefore, we investigated the pan-cancer relationship between TME and *FDX1* expression, using the ESTIMATE algorithm to calculate the stromal and immune cell scores in pan-cancer. Our results revealed that *FDX1* expression was significantly negatively correlated with immune and stromal scores in ACC, KIRC, STAD, and THCA and positively correlated in LGG and SARC (Supplementary Table S1). The immune scores were also significantly positively correlated with *FDX1* expression in BRCA and PCPG. The seven tumors with the highest correlation coefficients are presented in Figure 8, and results for the other cancers are shown in Supplementary Figure S3. These findings suggest that *FDX1* may influence the immune tolerance of tumors by regulating the TME.

### Relationship between *FDX1* expression and levels of tumor immune cell infiltration

**FIGURE 9:**
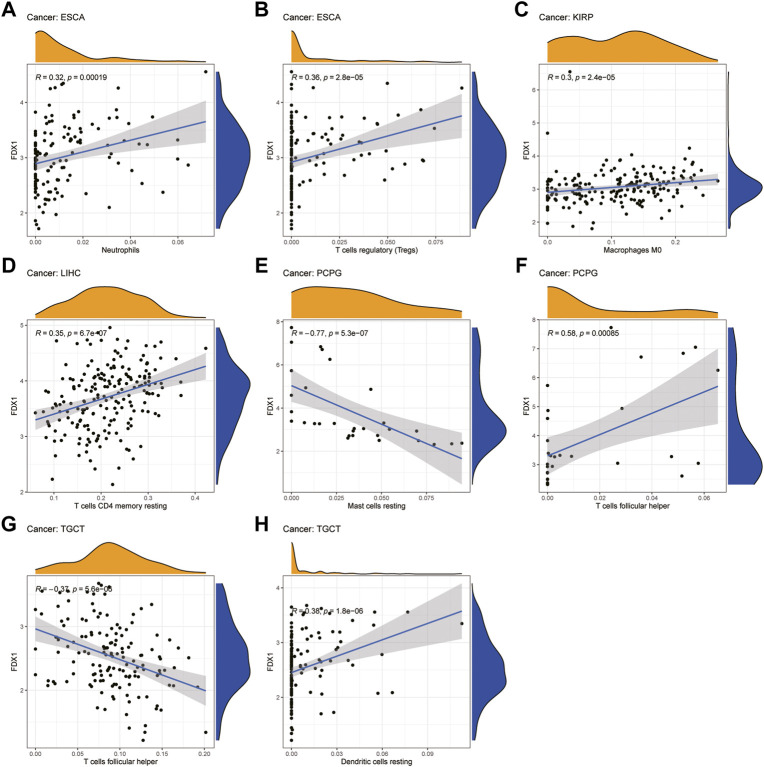
Relationship between *FDX1* expression and tumor infiltration of different immune cells in TCGA database. **(A–H)** Five tumors with the highest correlation coefficients between *FDX1* expression and immune-associated cell infiltration.

Next, we examined the relationship between *FDX1* expression and the levels of infiltration of 22 immune-related cells. Our results revealed that *FDX1* expression was positively correlated with the infiltration levels of macrophages M0, neutrophils, and mast cells activated and negatively correlated with mast cells resting in most cancers (Supplementary Table S1). For example, *FDX1* expression was positively correlated with the levels of infiltrating M0 macrophages in BRCA and KIRP. Likewise, the levels of infiltrating neutrophils were positively correlated with *FDX1* expression in ESCA and LGG. In contrast, *FDX1* expression was negatively correlated with the levels of mast cells resting in BRCA, LGG, and STAD, while it was positively correlated with mast cells activated in these cancers. We also analyzed the relationship between the expression of *FDX1* and the levels of infiltrating B cell, T cell, and NK cell and found that it had a negative correlation with the levels of B cells naive in LGG but positively in KIRC. Intriguingly, *FDX1* expression was negatively correlated with the levels of CD4 memory resting T cells in BRCA and positively in LGG and LIHC. Contrasting results showed that infiltrating follicular helper T cells levels were positively correlated with *FDX1* expression in BRCA and PCPG while negatively in LIHC and TGCT. In addition, the levels of infiltrating NK cells were significantly correlated with *FDX1* expression in SKCM, THCA, and TGCT. The five tumors with the highest correlation coefficients between the degree of infiltration and *FDX1* expression for each type of immune cell are presented in Figure 9, and the results for the other cancers are shown in Supplementary Figure S4. These results illustrate that *FDX1* may contribute to cancer immune escape by mediating tumor immune cell infiltration.

### Relationship between *FDX1* expression and expression levels of immune-related genes

**FIGURE 10:**
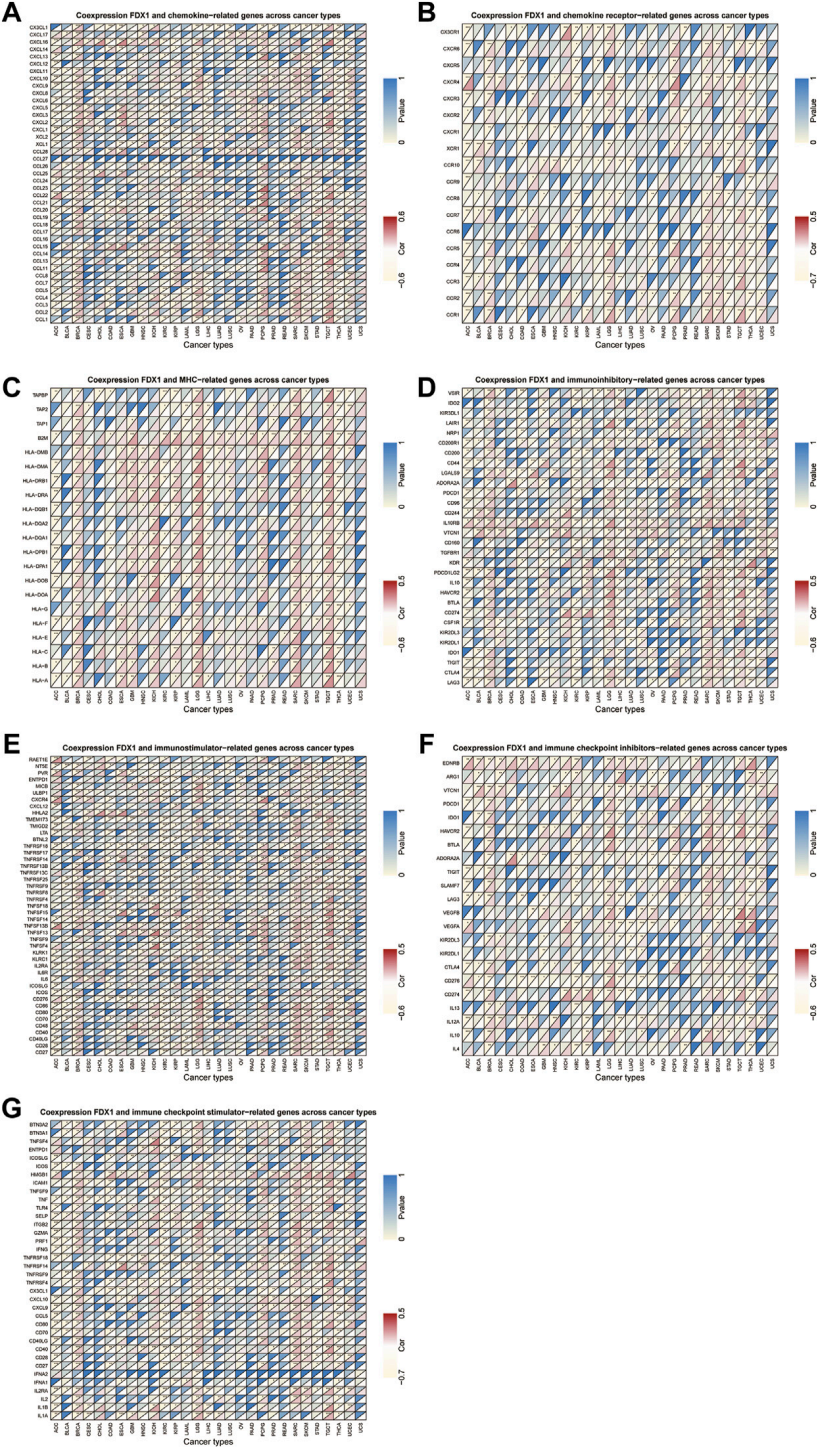
Relationship between FDX1 and **(A)** chemokine, **(B)** chemokine receptor, **(C)** major histocompatibility complex (MHC) genes, **(D)** immunosuppressive genes, **(E)** immunostimulatory genes, and **(F,G)** immune checkpoint-related genes. *p < 0.05, **p < 0.01, ***p < 0.001.

Next, we conducted gene co-expression analyses to explore the relationship between *FDX1* expression and immune-related genes, including chemokine, chemokine receptor, MHC, immunostimulatory, immunosuppressive, and immune checkpoint-related genes, in pan-cancer. Our data showed that expression of *CXCL16* (a chemokine-related gene) and *HLA-DMB* (an HLA-related gene) was positively correlated with *FDX1* expression in 13 tumors, excluding THCA (Figures 10A,C). Likewise, *CCR1* and *CD86* were negatively correlated with *FDX1* expression in ACC, LIHC, STAD, and THCA but were positively correlated in the other 9 tumors (Figures 10B,E). As shown in Figures 10D,F, *FDX1* expression was positively correlated with *IL10RB* (an immunosuppressive-related gene) and *HAVCR2* (an immune checkpoint inhibitor-related gene) in BRCA, GBM, HNSC, LGG, LUSC, SARC, SKCM, TGCT, and UCEC. In contrast, *FDX1* expression was negatively correlated with *CD40* expression in ACC, COAD, ESCA, KICH, KIRC, KIRP, acute myeloid leukemia (LAML), PRAD, STAD, and THCA, but was positively related to this gene expression in the other six cancers (Figure 10G, Supplementary Table S1). These results demonstrate that *FDX1* may promote tumor progression and immune escape by regulating immune-related genes.

### The prediction of the correlation between *FDX1* expression and drug sensitivity

**FIGURE 11:**
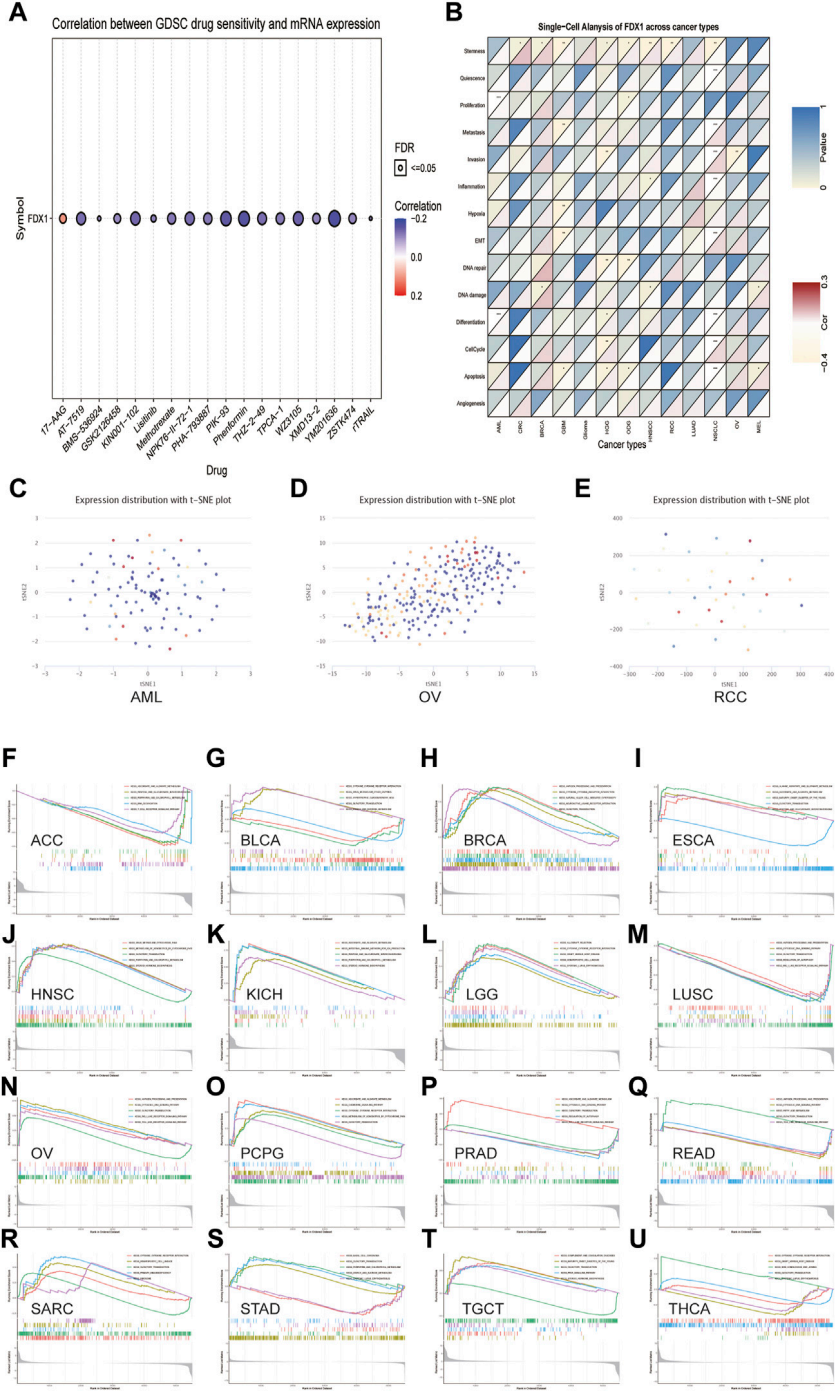
The relationship between *FDX1* mRNA expression and drug sensitivity and tumor functional status and its results of GSEA analysis. **(A)** Figure summarizes the correlation between *FDX1* expression and the top 30 sensitivity drugs across cancers based on the GDSC database. **(B)** Heatmap illustrating the correlation between *FDX1* expression and different tumor functional statuses based on the CancerSEA database. *p < 0.05, **p < 0.01, ***p < 0.001. **(C–E)** T-SNE diagram demonstrates *FDX1* expression profiles in single cells of AML, OV, and RCC samples, respectively. **(F–U)** KEGG pathway analysis of *FDX1* in multiple cancers. Curves of different colors show different functions or pathways regulated in different cancers. Peaks on the upward curve indicate positive regulation, and peaks on the downward curve indicate negative regulation.

The association between the anticancer drug sensitivity and *FDX1* mRNA expression was determined using the GDSC database, and *FDX1* expression was found to be significantly correlated with 42 drug responses (Supplementary Table S1). Our data showed that *FDX1* expression was negatively correlated with drug sensitivity in most cancers, such as AT−7519, KIN001−102, NPK76−II−72−1, PIK−93, Phenformin, and XMD13−2 (Figure 11A). In contrast, *FDX1* expression was positively correlated with sensitivity to two drugs or small molecules, including 17-AAG and CHIR-99021. These results indicate that *FDX1* is a potential therapeutic target in cancers.

### Expression pattern of *FDX1* in single cell and its relationship with cancer functional status

Single-cell transcriptomic sequencing is a crucial technique for analyzing diverse cancer cells, immune cells, endothelial cells, and stromal cells (Zhang et al., 2021b). To verify the *FDX1* expression level and its relationship with tumor functional status at the single-cell level in different cancers, we used the CancerSEA database. Our results revealed that *FDX1* expression was correlated with the functional state of stemness, invasion, differentiation, proliferation, metastasis, and DNA damage in several cancers (Figure 11B). For instance, *FDX1* expression was significantly positively correlated with differentiation and proliferation of AML and with stemness of renal cell carcinoma (RCC). In contrast, the invasion functional state of OV was significantly negatively correlated with *FDX1* expression. *FDX1* expression profiles are shown in single cells of AML, OV, and RCC using a T-SNE diagram (Figures 11C–E). These results demonstrate that *FDX1* participates in tumor development and metastasis.

### Gene set enrichment analysis of Ferredoxin 1

Next, we evaluated the pathway through which *FDX1* may be involved using GSEA in pan-cancer from TCGA and found that cytokine-cytokine receptor interaction and olfactory transduction pathways were closely correlated with *FDX1* expression in most tumors, which was significantly positively correlated with BRCA, PCPG, and SARC (Figures 11H, O, R). The results of GSEA showed that *FDX1* was predicted to be a negative regulator of the regulation of autophagy and RIG-I-like receptor signaling pathway in LUSC and PRAD and a positive regulator in OV (Figures 11M,P,N). Similarly, antigen processing and presentation and cytosolic DNA sensing pathway were positively regulated by *FDX1* in OV and acted as a negative regulator in LUSC and READ. As shown in Figures 11F,I,K, pentose and glucuronate interconversions pathway was negatively correlated with *FDX1* expression in ACC but positively correlated with ESCA and KICH. The results for the other cancers are shown in Figures 11 G,J,L,Q,S–U. In summary, these results suggest that *FDX1* plays an essential role in tumor immunity and development.

### Protein-protein interaction network of *FDX1*

We created a PPI network for *FDX1* using STRING to identify probable processes by which *FDX1* contributes to carcinogenesis. Supplementary Figure S5 shows that *FDX1* is closely associated with Fe-S proteins such as heat shock cognate B mitochondrial iron-sulfur cluster cochaperone (*HSCB*), iron-sulfur cluster assembly enzyme (*ISCU*), and iron-sulfur cluster assembly 1 (*ISCA1*) and cytochrome P450 proteins such as cytochrome P450 family 11 subfamily A member 1 (*CYP11A1*) and cytochrome P450 family 11 subfamily B member 2 (*CYP11B2*).

### Ferredoxin 1 is downregulated by elesclomol, resulting in inhibiting of cell viability in bladder urothelial carcinoma, clear cell renal cell carcinoma, and prostate cancer

**FIGURE 12:**
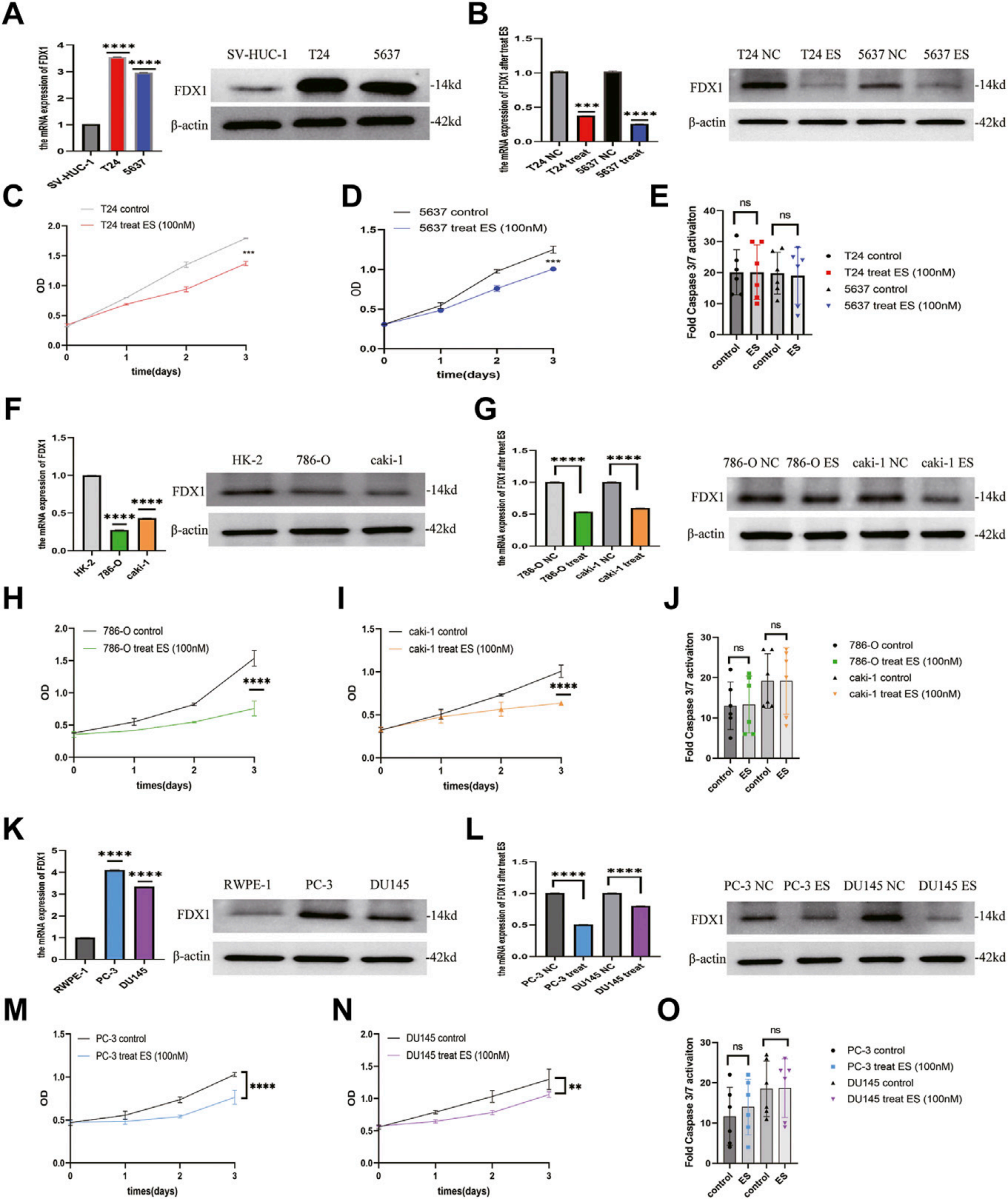
FDX1 is downregulated by elesclomol, resulting in inhibiting cell viability in BLCA, ccRCC, and PCa. **(A,F,K)** The expression level of *FDX1* was evaluated in BLCA, ccRCC, and PCa cells and their corresponding normal cells by qRT-PCR and western blot. **(B,G,L)** The expression level of *FDX1* in BLCA, ccRCC, and PCa cell lines treated with elesclomol after 16 h. **(E,J,O)** The fold Caspase 3/7 activity in BLCA, ccRCC, and PCa cell lines after treated with elesclomol after 16 h. **(C,D,H,I,M,N)** The cell viability of BLCA, ccRCC, and PCa cell lines after treatment with elesclomol.

To verify the results of the above analysis, we firstly examined the expression of *FDX1* in BLCA, ccRCC, and PCa cell lines. As shown in Figures 12A,F,K, relatively higher expression of *FDX1* was detected in T24 and 5637 cells than in SV-HUC-1 cells. FDX1 was highly expressed in PC-3 and DU145 cells than in RWPE-1 cells. In contrast, *FDX1* has low expression in 786-O and caki-1 cells than in HK-2 cells. According to the previous research (Tsvetkov et al., 2022), elesclomol directly targeted *FDX1* and promoted copper-dependent cell death. Thus, we performed a CCK-8 assay to evaluate the effect of elesclomol on BLCA, ccRCC, and PCa cells proliferation. The CCK-8 assay showed that the cell viability of the elesclomol treatment group was markedly lower than that of the control group (Figures 12C,D,H,I,M,N). Moreover, downregulation of *FDX1* was detected in the elesclomol treatment group (Figures 12B,G,L). We further examined the effect of elesclomol on caspase-3/7 activity. Our results showed that caspase-3/7 activity did not change in the elesclomol treatment group relative to the control group (Figures 12E,J,O). These results indicate that *FDX1* is downregulated by elesclomol, inhibiting cell viability *in vitro*, without activating caspases 3 and 7.

## Discussion

The pan-cancer analysis provides a comprehensive understanding of the molecular aberrations and functional roles across various cancers. It helps to identify new diagnostic biomarkers and new therapeutic targets for cancers (Weinstein et al., 2013). FDX1 functions by transferring electrons from NADPH to mitochondrial cytochrome P450 via the ferredoxin reductase (Sheftel et al., 2010) and is a crucial regulator of copper-induced cell death (Tsvetkov et al., 2022). Accumulating evidence indicated that *FDX1* was closely related to the occurrence and development of various tumors (Zhang et al., 2008; Zhang et al., 2017; Zhang et al., 2020). However, *FDX1* has not mainly been elucidated in cancer, and its role in tumorigenesis or pan-cancer is still unclear. Therefore, in our study, we conducted a pan-cancer analysis of *FDX1* in different cancers based on the data from the most comprehensive databases and also explored the effect of *FDX1* in BLCA, ccRCC, and PCa cells for the first time.

We first assessed the expression and prognostic significance of *FDX1* in pan-cancer. Our results showed that *FDX1* was dysregulated in 17 types of cancer, and IHC analysis confirmed this tendency at the protein level in thyroid cancer, colorectal cancer, and liver cancer. Our research reached the opposite result to previous studies when we used only TCGA database analysis. However, when we analyzed by combining TCGA and GTEx databases, we drew identical results to those of previous research (Zhang et al., 2008; Tsvetkov et al., 2019; Zhang et al., 2021a). For example, knockdown of *FDX1* neither inhibited tumor cell growth nor induced apoptosis but inhibited the ATP production and fatty acid oxidation in LUAD cells (Zhang et al., 2021a). FDX1 is highly expressed in human malignant melanoma cells, which correlates with resistance to apoptosis induced by ultraviolet treatment (Zhang et al., 2008). Elesclomol directly targets *FDX1* in human breast cancer and lung adenocarcinoma cells, inhibits *FDX1*-mediated Fe-S cluster biosynthesis, and promotes copper-dependent cell death (Tsvetkov et al., 2019). This discrepancy may be due to differences in tumor samples or the limited number of normal samples for some cancers in the TCGA database. Regarding *FDX1* mRNA expression and its protein expression are not completely consistent, which may be due to temporal and spatial gaps between the transcription and translation in eukaryotic gene expression. Meanwhile, there are many modifications in transcriptional and post-translational, as supported by previous research (Gu et al., 2009). Notably, the overexpression of *FDX1* was correlated with better prognosis in KIRC, THCA, and LIHC but was the opposite in ACC, HNSC, LGG, and PAAD. These findings demonstrated that *FDX1* could be used as a biomarker of the prognosis for various cancers.

Both DNA methylation alterations and RNA methylation modification play crucial roles in tumorigenesis (Klutstein et al., 2016; Zhao et al., 2017). Our study revealed that *FDX1* expression was significantly correlated with DNA methylation in six tumors, with positive correlations in BRCA, KIRP, LIHC, and LUSC but negative correlations in BLCA and KIRC. Furthermore, in most cancers, *FDX1* expression was significantly positively correlated with m6A-, m5C-, and m1A-related genes, such as *YTHDF1*, *YTHDF2*, *TRMT10C*, and *NSUN6*. These findings suggest that the changes in the epigenetic status of *FDX1* may contribute to tumorigenesis. Tumors with defects in the MMR system will cause high levels of MSI, which leads to the aggravation of TMB and results in tumor occurrence (Picard et al., 2020). Our results illustrated that *FDX1* expression was highly correlated with MMR gene, MSI, and TMB in most cancers. These results showed the function of *FDX1* in mediating tumorigenesis by regulating DNA and RNA methylation, MMR gene, MSI, and TMB, which was consistent with the previous studies (Klutstein et al., 2016; Zhao et al., 2017; Picard et al., 2020).

Our results showed that *FDX1* is also strongly involved in cancer immunity. TME plays a decisive role in tumor initiation, progression, and response to therapies (Xiao and Yu, 2021). According to the ESTIMATE scores, there was a negative correlation between *FDX1* expression and stromal and immune cell content in the TME of ACC, KIRC, STAD, and THCA, but not in LGG and SARC. Tumor-infiltrating immune cells contribute significantly to the homeostasis of TME and play a vital role in the occurrence, development, and immunotherapy of tumors (Lei et al., 2020). As members of the tumor-infiltrating immune cells, mast cells and natural killer cells may either suppress cancer or support tumor growth (Costa et al., 2021). Our data showed that *FDX1* had a negative relationship with NK cells activated in most cancers. This finding may explain the risk role of *FDX1* in most tumor types. Furthermore, our study also revealed the co-expression of *FDX1* with immune-related genes, including chemokine, chemokine receptor, MHC, immunostimulatory, immunosuppressive, and immune checkpoint-related genes. *FDX1* expression is closely correlated with almost all immune-related genes and contributes to tumor development (Li et al., 2021; Schaafsma et al., 2021). Overall, these results indicate that the regulation of *FDX1* expression may be a promising strategy to increase the efficacy of immunotherapy.

Mounting evidence demonstrated that some new advances had been made in cancer treatment, such as RNA interference (Tan et al., 2010), targeting of the ubiquitin-proteasome pathway (Narayanan et al., 2020), and targeting of tumor suppressor genes (Gao et al., 2021). However, drug resistance is a major obstacle to pre-clinical and clinical therapies. We analyzed the correlation between *FDX1* expression and IC_50_ of over 750 anticancer drugs. The results suggested that *FDX1* expression was closely related to sensitivity of many drugs, but high expression of *FDX1* reduced the sensitivity to 17-AAG and CHIR-99021. This phenomenon means that its potential role in drug resistance requires further research. However, we reached the opposite result to a previous study, in which elesclomol could directly target *FDX1* and promote copper-induced cell death in human rhabdomyosarcoma and LUAD cells (Tsvetkov et al., 2022). This discrepancy may be due to differences in tumor cell lines, as previous studies have focused more on cells with high metastasis. This finding suggests that the role of *FDX1* in copper-induced cell death requires further investigation.

Next, we used the PPI network, the CancerSEA database, and GSEA analysis to address the function of *FDX1* in pan-cancer specifically. The PPI network showed that *FDX1* interacted with *HSCB*, *ISCU*, *ISCA1*, *CYP11A1*, and *CYP11B2*, all associated with the mitochondrial respiratory chain. The results based on the CancerSEA database showed that *FDX1* is closely related to differentiation, proliferation, stemness, and invasion at the single-cell level in most cancers. Consistent with our data, *FDX1* was previously reported to be involved in cell proliferation (Zhang et al., 2020) and thus resulted in cancer development. Furthermore, *FDX1* can inhibit copper-induced cell death in LUAD cell lines without the involvement of apoptosis-related genes (Tsvetkov et al., 2019; Tsvetkov et al., 2022). The KEGG pathway analysis demonstrated that *FDX1* was significantly correlated with the pathway of pentose and glucuronate interconversions, cytokine-cytokine receptor interaction, and regulation of autophagy. Supporting that, *FDX1* also plays a vital role in glucose metabolism (Zhang et al., 2021a), promoting tumor progression.

Furthermore, we performed a series of *in vitro* experiments to investigate the effect of *FDX1* on BLCA, ccRCC, and PCa cells. These analyses demonstrated a relatively higher expression of *FDX1* in BLCA and PCa cell lines and low expression in ccRCC cells, consistent with our bioinformatic analytical result. In addition, elesclomol inhibited cell viability *in vitro* without activating caspases 3 and 7 and decreased the expression of *FDX1*, which was consistent with findings of a previous study (Tsvetkov et al., 2019; Tsvetkov et al., 2022). These results indicate that *FDX1* is downregulated by elesclomol, which inhibited BLCA, ccRCC, and PCa cells viability *in vitro* without activating caspases 3 and 7.

Our study had several limitations. First, some contradictory findings regarding individual cancers were observed in our study. Therefore, it is necessary to investigate the expression and function of *FDX1* further using a larger sample size. Second, our findings suggested that *FDX1* can serve as a prognostic factor for different tumors, which requires further verification. Third, the effects of *FDX1* on the tumor microenvironment and immunotherapy required experimental and clinical validation. Fourth, although we confirmed that elesclomol targeted *FDX1* and inhibited cell viability of BLCA, ccRCC, and PCa cells, the precise regulatory mechanisms remain unclear and require further exploration.

In summary, our first pan-cancer analysis of *FDX1* indicated that this factor was differentially expressed between tumor and normal tissues and revealed correlations between *FDX1* expression and DNA methylation and RNA methylation-related genes. Our findings suggested that *FDX1* may be a prognostic factor for different tumors. Moreover, *FDX1* expression was associated with MMR gene, MSI, TMB, and immune cell infiltration in different cancer types. Its impact on tumor immunity and drug sensitivity also varied with tumor types. Importantly, elesclomol targeted *FDX1* and inhibited cell viability in BLCA, ccRCC, and PCa cells. These findings may help clarify the role of *FDX1* in tumorigenesis and development and provide a reference for more accurate and personalized immunotherapy in the future.

## Data Availability

The original contributions presented in the study are included in the article/Supplementary Material, further inquiries can be directed to the corresponding authors.

## Glossary

FDX1: Ferredoxin 1
NADPH: Nicotinamide Adenine Dinucleotide Phosphate Hydrogen
TCGA: The Cancer Genome Atlas
CCLE: Cancer Cell Line Encyclopedia
GTEx: Genotype Tissue-Expression;
HPA: Human Protein Atlas
GSCA: Gene Set Cancer Analysis
MMR: mismatch repair
MSI: microsatellite instability
TMB: tumor mutation burden
TME: tumor microenvironment
PD-1: programmed cell death protein-1
PD-L1: programmed cell death 1 ligand 1
CTLA4: cytotoxic T-lymphocyte associated protein 4
β2MG: β2-microglobulin
HLA: human leukocyte antigen
Fe-S: iron-sulfur
SF-1: steroidogenic factor-1
PCOS: polycystic ovary syndrome
LPD: Lymphoproliferative disorders
IgA: Immunoglobulin A
ES: elesclomol
UCSC: University of California SANTA CRUZ
RNA: Ribonucleic Acid
IHC: immunohistochemistry
mRNA: Messenger RNA
DNA: Deoxyribonucleic acid
m6A: N6-methyladenosine
m5C: methyl-5-cytosine
m1A: N1-methyladenosine
CBLL1: Cbl proto-oncogene like 1
WTAP: WT1 associated protein
METTL3: methyltransferase 3 N6-adenosine-methyltransferase complex catalytic subunit
METTL14: methyltransferase 14 N6-adenosine-methyltransferase complex catalytic subunit
RBM15: RNA binding motif protein 15
RBM15B: RNA binding motif protein 15 beta
ZC3H13: zinc finger CCCH-type containing 13
ALKBH5: alkB homolog 5, RNA demethylase
FTO: FTO alpha-ketoglutarate dependent dioxygenase
IGF2BP1: insulin like growth factor 2 mRNA binding protein 1
YTHDC1: YTH domain containing 1
YTHDC12: YTH domain containing 2
YTHDF1: YTH N6-methyladenosine RNA binding protein 1
THDF2: YTH N6-methyladenosine RNA binding protein 2
YTHDF3: YTH N6-methyladenosine RNA binding protein 3
NSUN7: NOP2/Sun RNA methyltransferase family member 7
NSUN2: NOP2/Sun RNA methyltransferase 2
NSUN3: NOP2/Sun RNA methyltransferase 3
NSUN4: NOP2/Sun RNA methyltransferase 4
NSUN5: NOP2/Sun RNA methyltransferase 5
NSUN6: NOP2/Sun RNA methyltransferase 6
TRDMT1: tRNA aspartic acid methyltransferase 1
DNMT1: DNA methyltransferase 1
DNMT3: DNA methyltransferase 3
DNMT3A: DNA methyltransferase 3 alpha
DNMT3B: DNA methyltransferase 3 beta
NOP2: NOP2 nucleolar protein
TET2: tet methylcytosine dioxygenase 2
TRMT6: tRNA methyltransferase 6 non-catalytic subunit
ALYREF: Aly/REF export factor
TRMT61A: tRNA methyltransferase 61A
TRMT61B: tRNA methyltransferase 61B
ALKBH1: alkB homolog 1, histone H2A dioxygenase
ALKBH3: alkB homolog 3, alpha-ketoglutarate dependent dioxygenase
KM: Kaplan-Meier
OS: overall survival
DSS: disease-specific survival
DFI: disease-free interval
PFI: progression-free interval
MLH1: mutL homolog 1
MSH2: mutS homolog 2
MSH3: mutS homolog 3
MSH4: mutS homolog 4
MSH5: mutS homolog 5
MSH6: mutS homolog 6
PMS1: PMS1 homolog 1, mismatch repair system component
PMS2: PMS1 homolog 2, mismatch repair system component
ESTIMATE: Estimation of STromal and Immune cells in MAlignant Tumor tissues using Expression data
CIBERSORT: Cell-type Identification By Estimating Relative Subsets Of RNA Transcripts
MHC: major histocompatibility complex
GDSC: Genomics of Drug Sensitivity in Cancer
CancerSEA: Cancer Single-cell State Atlas
t-SNE: t-distributed Stochastic Neighbor Embedding
GSEA: Gene Set Enrichment Analysis
KEGG: Kyoto Encyclopedia of Genes and Genomes
PPI: Protein-Protein Interaction
ccRCC: Clear cell renal cell carcinoma
PCa: Prostate cancer
FBS: fetal bovine serum
DMEM: dulbecco’s modified eagle medium
RPMI: Roswell Park Memorial Institute
RT: reverse transcription
RT-qPCR: Reverse Transcription-Quantitative Polymerase Chain Reaction
cDNA: complementary DNA
RIPA: Radio Immunoprecipitation Assay
BCA: bicinchoninic acid
SDS-PAGE: sodium dodecyl sulfate-polyacrylamide gel electrophoresis
PVDF: polyvinylidene fluoride
CCK-8: Cell Counting Kit-8
OD: optical density
log2: logarithm2
ACC: Adrenocortical carcinoma
BLCA: Bladder Urothelial Carcinoma
BRCA: Breast invasive carcinoma
CESC: Cervical squamous cell carcinoma and endocervical adenocarcinoma
CHOL: Cholangiocarcinoma
COAD: Colon adenocarcinoma
DLBC: Lymphoid Neoplasm Diffuse Large B-cell Lymphoma
ESCA: Esophageal carcinoma
GBM: Glioblastoma multiforme
HNSC: Head and Neck squamous cell carcinoma
KICH: Kidney Chromophobe
KIRC: Kidney renal clear cell carcinoma
KIRP: Kidney renal papillary cell carcinoma
LAML: Acute Myeloid Leukemia
LGG: Brain Lower Grade Glioma
LIHC: Liver hepatocellular carcinoma
LUAD: Lung adenocarcinoma
LUSC: Lung squamous cell carcinoma
MESO: Mesothelioma
OV: Ovarian serous cystadenocarcinoma
PAAD: Pancreatic adenocarcinoma
PCPG: Pheochromocytoma and Paraganglioma
PRAD: Prostate adenocarcinoma
READ: Rectum adenocarcinoma
SARC: Sarcoma
SKCM: Skin Cutaneous Melanoma
STAD: Stomach adenocarcinoma
TGCT: Testicular Germ Cell Tumors
THCA: Thyroid carcinoma
THYM: Thymoma
UCEC: Uterine Corpus Endometrial Carcinoma
UCS: Uterine Carcinosarcoma
UVM: Uveal Melanoma
M0: Membranous 0
B cell: B Lymphocyte
T cell: thymus dependent lymphocyte
NK: natural killer cell
CD4: cluster of differentiation 4
CXCL16: C-X-C motif chemokine ligand 16
HLA-DMB: major histocompatibility complex, class II, DM beta
CCR1: C-C motif chemokine receptor 1
CD86: cluster of differentiation 86
IL10RB: interleukin 10 receptor subunit beta
HAVCR2: hepatitis A virus cellular receptor 2
CD40: cluster of differentiation 40
AML: acute myeloid leukemia
RCC: renal cell carcinoma
RIG-I-like: Retinoic acid-inducible gene I-like
HSCB: iron-sulfur cluster cochaperone
ISCU: iron-sulfur cluster assembly enzyme
ISCA1: iron-sulfur cluster assembly 1
CYP11A1: cytochrome P450 family 11 subfamily A member 1
CYP11B2: cytochrome P450 family 11 subfamily B member 2
ATP: Adenosine triphosphate
IC_50_: half maximal inhibitory concentration.
