# Supplementary material for: Ferredoxin 1 is a cuproptosis-key gene responsible for tumor immunity and drug sensitivity: A pan-cancer analysis
Source: Front Pharmacol. 2022 Sep 21;13:938134. doi: 10.3389/fphar.2022.938134 (PMC9532935; doi:10.3389/fphar.2022.938134)
Supplement: Supplementary file 1 [file DataSheet1.PDF]

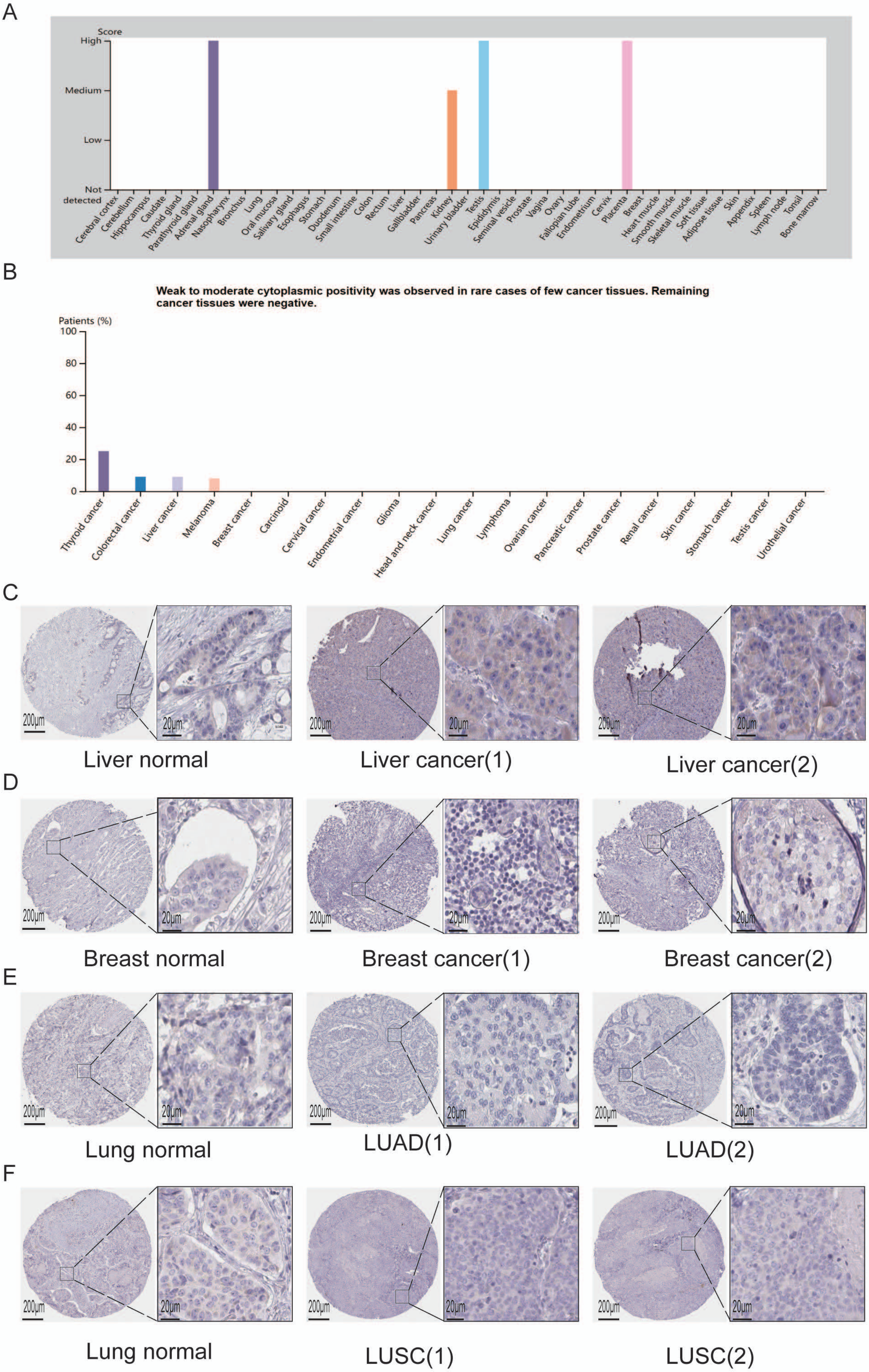

Supplemental FIGURE 1. The protein expression of FDX1 in different normal and tumor tissues and its immunohistochemistry images of normal and tumor tissues were from the HPA database. (A,B) Represent the protein expression of FDX1 in different normal and tumor tissues. (B-E) Represent immunohistochemical images of FDX1 protein expression in normal (left) and tumor (right) tissues of the liver, breast, and lung, respectively.

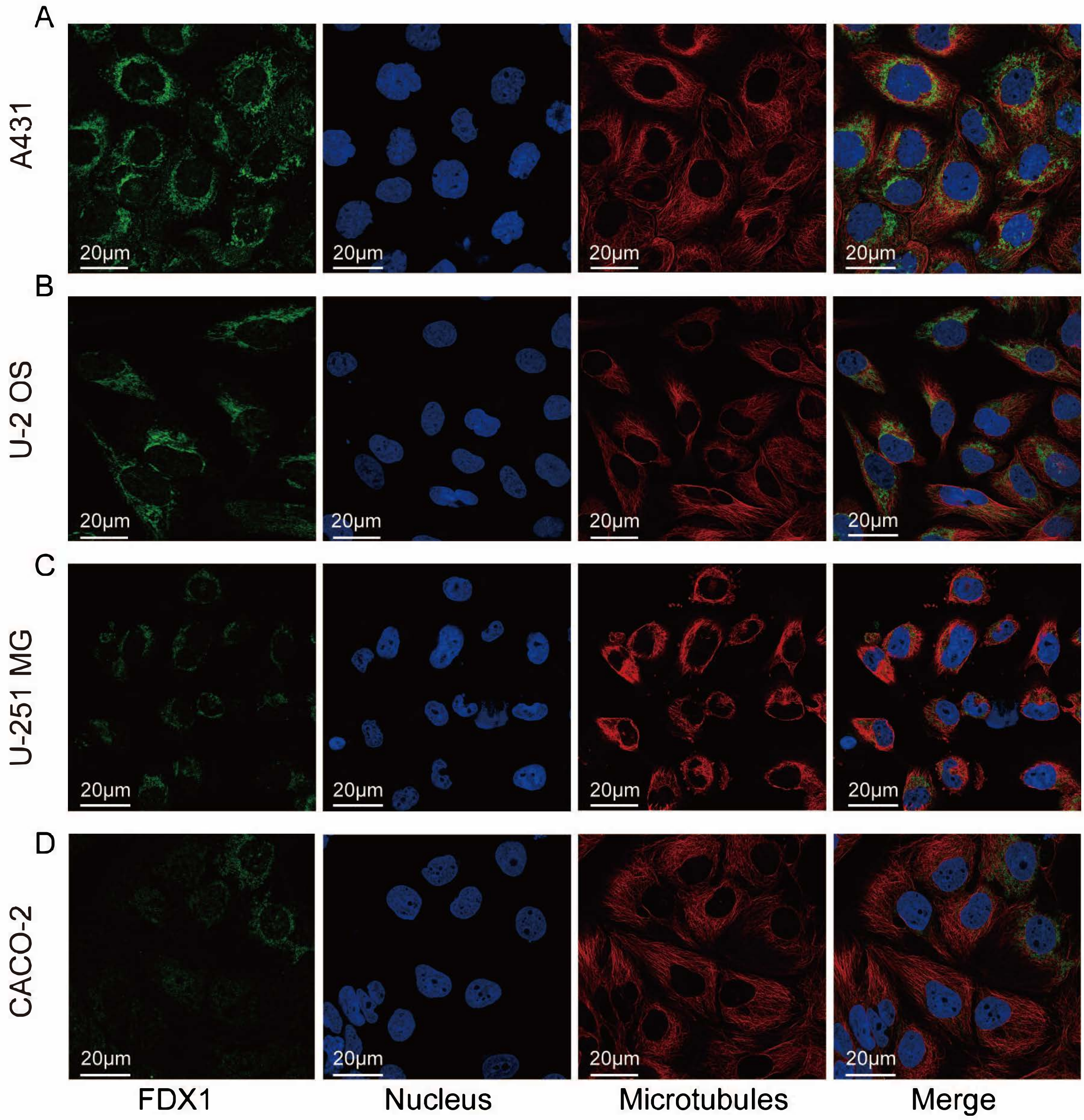

Supplemental FIGURE 2. The subcellular location of the protein of *FDX1* in different cancer cells was from the HPA database. (A-D) Represent the subcellular localization of *FDX1* in cell lines of A-431, U-2 OS, U-251 MG, and CACO-2, respectively.

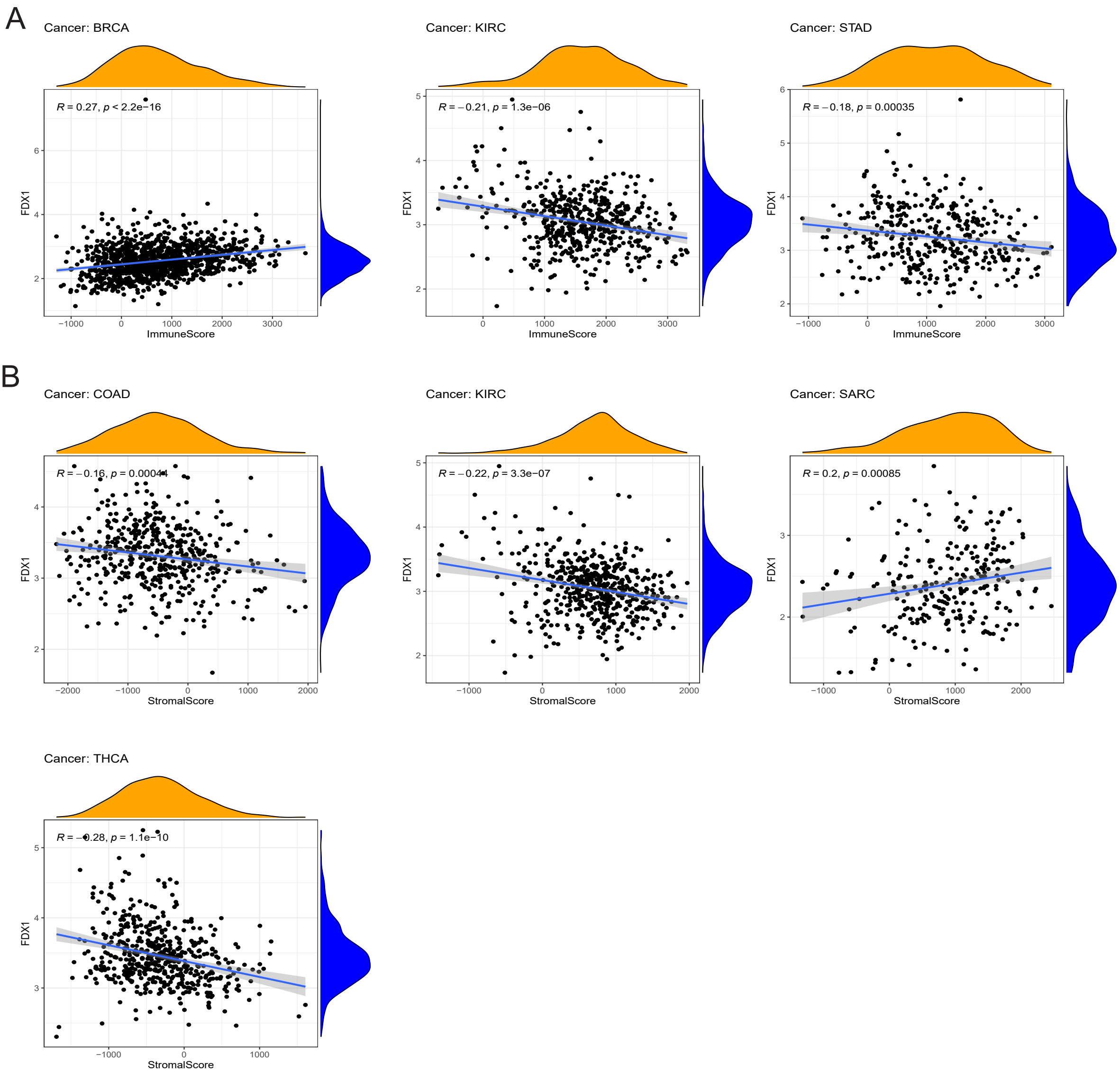

Supplemental FIGURE 3. Six tumors with the correlation coefficients between *FDX1* expression and the tumor microenvironment. (A) Correlation between *FDX1* and immune scores in BRCA, KIRC, and STAD. (B) Correlation between *FDX1* and stromal scores in COAD, KIRC, SARC, and THCA.

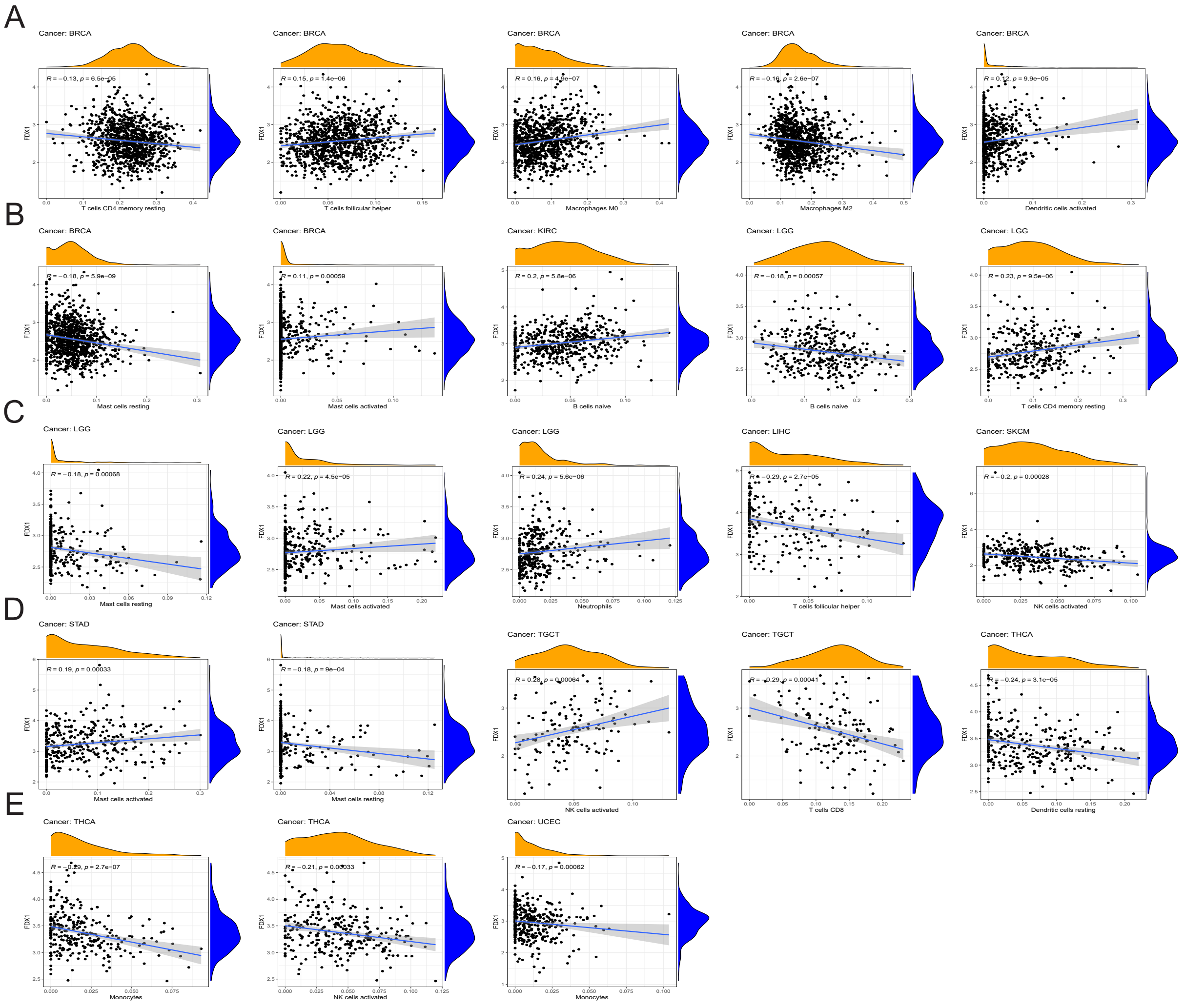

Supplemental FIGURE 4. Nine tumors with the correlation coefficients between *FDX1* expression and tumor infiltration of different immune cells in the TCGA database.

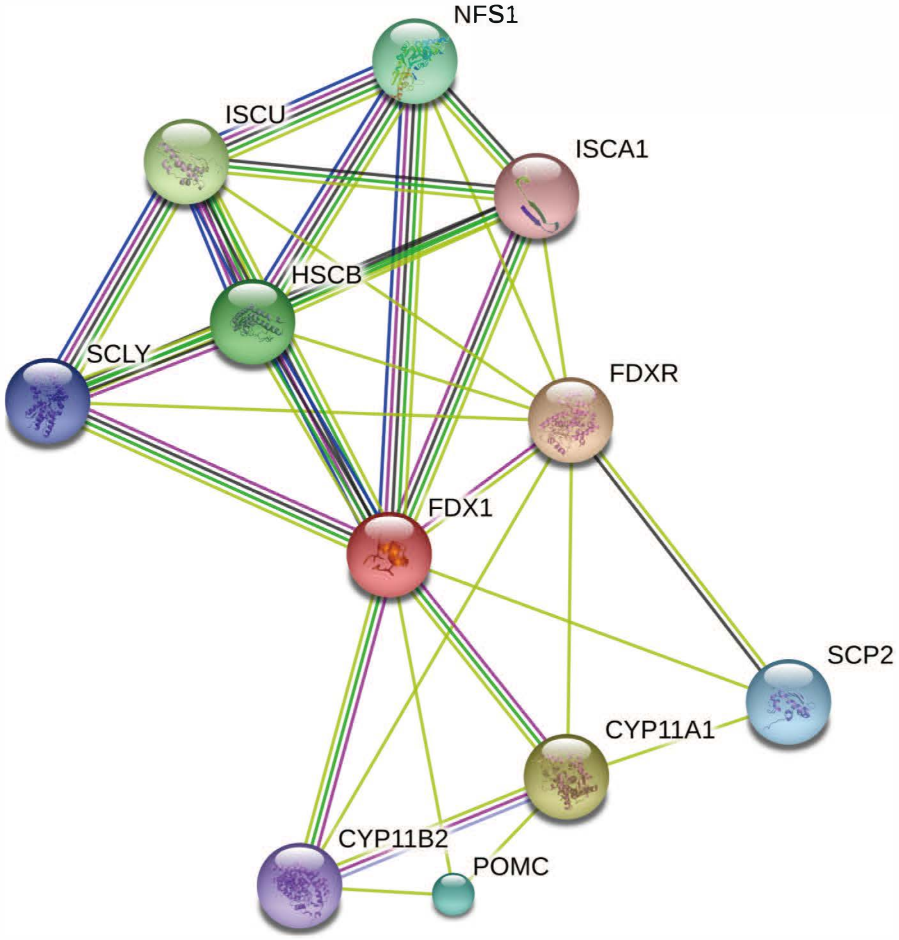

Supplemental FIGURE 5. Available determined *FDX1*-binding proteins was [illegible] obtained with the STRING tool.
